# Textural effect of Pt catalyst layers with different carbon supports on internal oxygen diffusion during oxygen reduction reaction

**DOI:** 10.3389/fchem.2023.1217565

**Published:** 2023-06-07

**Authors:** Wenli Zhang, Zhejie Ma, Xuankai Zhao, Liheng Zhou, Liu Yang, Ping Li

**Affiliations:** State Key Laboratory of Chemical Engineering, East China University of Science and Technology, Shanghai, China

**Keywords:** oxygen reduction reaction, catalyst layer structure, carbon support, oxygen diffusion coefficient, rotating disk electrode

## Abstract

One way to address the cost issue of polymer electrolyte membrane fuel cells (PEMFCs) is to reduce the amount of platinum used in the cathode catalyst layers (CLs). The oxygen mass transfer resistance of the cathode CLs is the main bottleneck limiting the polarization performance of low Pt-loading membrane electrodes at high current densities. Pt nanoparticles, ionomers, carbon supports, and water in cathode CLs can all affect their oxygen mass transfer resistance. From the perspective of carbon supports, this paper changed the texture of CLs by adding carbon nanotubes (CNTs) or graphene oxide (GO) into carbon black (XC72) and studied its impact on the oxygen mass transfer resistance. A mathematical model was adopted to correlate total mass transfer resistance and internal diffusion efficiency factor with CL structure parameters in order to determine the dominant textural effect of a CL. The results show that adding 30%CNT or 20GO to carbon black of XC72 improved the electrocatalytic performance and mass transfer capability of the composite carbon-supported Pt catalyst layers during oxygen reduction reaction. The study further reveals that the smaller particle-sized carbon material with tiny Pt nanoparticles deposition can minimize the internal oxygen diffusion resistance. A less dense CL structure can reduce the oxygen transfer resistance through the secondary pores. The conclusion obtained can provide guidance for the rational design of optimal cathode CLs of PEMFCs.

## 1 Introduction

Under the international context of pursuing carbon neutrality, proton exchange membrane fuel cells (PEMFCs) are expected to be a new generation of green, high-efficient and stable power supplies that use carbon-free hydrogen as fuel rather than conventional fossil fuels (Das et al., 2017; Xing et al., 2019; Yang et al., 2022). For a successful commercialization of PEMFCs technology, however, an urgent need to be solved is to reduce its manufacturing costs, especially the cost of catalysts, by using super low Pt-loading and even non-Pt catalysts (Ganesan and Narayanasamy, 2019; Wang et al., 2019). In order to meet both the efficiency and power goals of fuel cell vehicles, it is essential to maintain high performance at high current density. A series of alloys and core-shell catalysts with low Pt-loading and high oxygen reduction reaction activity at low current density have been developed. Unfortunately, the approaches of reducing Pt-loading amount can bring about an increased oxygen transport resistance for oxygen reduction reaction (ORR) on Pt-based cathode catalyst layers (CLs), resulting in a significant performance loss at high current density (Chen et al., 2020; Cheng et al., 2022). Kongkanand and Mathias found that when the Pt-loading was reduced from 0.2 mg cm^-2^ to 0.05 mg cm^-2^, the voltage drop of the cell in the low current density region (0.2 A cm^-2^) was about 40 mV. However, it exceeded 200 mV in the high current density region (2 A cm^-2^), which is unacceptable in practical applications (Kongkanand and Mathias, 2016). Therefore, exploring how to enhance the mass transfer process of oxygen in CLs is critical for improving PEMFC performance with low Pt-loadings, mainly when operated in a high current density output region.

A typical CL is constructed out of ionomer, carbon particles, and active metal nanoparticles, which agglomerate together, forming the texture of the CL (Lin et al., 2021; Li et al., 2022). The carbon particles play the role of electron transportation and dispersion of active metal nanoparticles (Lin et al., 2019). Carbon black is a commonly used carbon support material for PEMFC, with its primary carbon particles having a size of 20–200 nm in diameter. Although few pores open on the surface of carbon black particles, many mesoporous voids exist inside and outside the particle agglomerates, becoming the channels for mass transfer throughout the CL (Holdcroft, 2013). From this point of view, intensifying the mass transfer should start by modifying the CL texture by modulating the agglomeration state of carbon black particles.

Porosity and pore size distribution are quantitative parameters for judging the CL texture (Abraham and Murugavel, 2021; Xi et al., 2022). Zhao et al. characterized CL pore structure by the method of standard porosimetry. They found that for the same type of catalysts, the porosity and mean pore size decreased when the Pt-loading was increased, while the pore surface area enlarged (Zhao et al., 2018). Ramaswamy et al. investigated the effect of the microstructure of carbon supports on the mass transfer properties at high current densities by measuring the oxygen local transport resistance with limiting current and H_2_/N_2_ impedance tests. They found that the local-oxygen transport and bulk-proton transport resistances were directly related to the micropore and macropore of carbon supports (Ramaswamy and Kumaraguru, 2018).

Mathematical models are practical tools that can calculate process details beyond the existing experimental capabilities to quantify the influence of CL texture on oxygen transport resistance. Chen et al. (2018) used pore-scale simulation to study oxygen transportation through four-constituent microscopic structures of the catalyst layer, including carbon particles, ionomer, Pt particles, and the primary pores embedded in the carbon particles. The simulation results showed that the local transport resistance could be reduced by depositing more Pt particles outside the carbon particles and relieving carbon particle agglomeration. He et al. (2020) established a comprehensive three-dimensional multiphase non-isothermal model coupled with an improved electrochemical kinetics model considering the geometric structure parameters of cathode CL and the oxygen transport process in CL. They found that the oxygen concentration on the Pt particles increased significantly with the decrease of carbon particle radius. The agglomerate model can simulate the influence of catalyst layer structure details on PEMFC performance and can thoroughly depict the reactants’ mass transfer process. Xie et al. developed an improved agglomerate sub-model of CL involving reasonable agglomerate size and Pt loading effect on oxygen transport. The local oxygen transport characteristics were elaborated by analyzing the oxygen transport resistance on a single aggregate. They revealed that the local transport resistance mainly originates from the Pt/carbon agglomeration and ionomer coverage (Xie et al., 2019). Our previous study also explored the influences on both the nominal-working and cold-start performance of PEMFC from the aspects of the catalyst layer structure and carbon material properties based on the agglomerate model (Yang et al., 2022; Yang et al., 2023). We found that the catalyst layer thickness, the carbon particle radius, and the platinum fraction inside carbon micro-pores play a critical role in the cell performance under the overall working conditions.

On the other hand, researchers endeavor all the time to find new carbon supports that can improve catalytic activity and stability as well as mass transfer ability, such as carbon nanofibers (Álvarez et al., 2012; Tuo et al., 2016), carbon nanotubes (CNTs) (Litkohi et al., 2020), carbon gels (Labbé et al., 2019). CNTs have been extensively adopted as supports for Pt catalysts due to their unique properties, including high surface area and good electrical conductivity. Meanwhile, CNTs are featured in high aspect ratios, which can form staggered networks of pores. Murata et al. (2014) developed CNTs-based cathode electrodes with anti-agglomeration properties on stainless steel substrates. They believed that the structure could improve the performance and optimize the transform route for oxygen, proton, electron, and water at high current density. The reason was that the CNTs were not agglomerated like carbon black so that the catalyst layer could contact well with the microporous layer, and the pores in the catalyst layer were perforative, which could benefit the diffusion of oxygen. Graphene is another new generation of carbon materials, possessing excellent thermal conductivity, high carrier mobility and high electrical conductivity. Kou et al. studied the electrocatalytic effect of Pt nanoparticles supported on functionalized graphene sheet (FGS) for ORR. Compared with commercial catalysts, Pt-FGS had higher electrochemical specific surface area and ORR activity and better stability. The characterization results indicated that the improved performance of Pt-FGS could be attributed to smaller Pt particles and less agglomeration (Kou et al., 2009).

Although various new carbon materials have expanded their applications to PEMFCs catalyst systems, more study needs to be paid attention to a quantitative description and comparison of their textural influences on oxygen diffusivity in CL. In the present study, we first prepared two series of carbon composite supports for Pt catalyst by mixing functionalized CNTs or graphene material with commonly used Vulcan XC72 carbon black particles, forming two kinds of catalyst layer textures with different porosities and tortuosities. Electrochemical properties of the supported Pt catalysts were then tested during ORR using a rotating disk electrode (RDE) apparatus, supplemented by the calculation and verification of the agglomerate model. The internal diffusion efficiency factors of various Pt catalysts supported on different carbon materials were compared, aiming to provide guidance for constructing a more reasonable catalyst layer structure.

## 2 Experimental

### 2.1 Materials and chemicals

The raw materials and chemicals used were PK-XC72 catalyst (40 wt. %Pt, Premetek), polydiallyldimethylammonium chloride (20 wt.% PDDA aqueous solution, Sigma-Aldrich), Nafion solution (5 wt.% in lower aliphatic alcohols and water, Sigma-Aldrich), and ethylene glycol (EG) and chloroplatinic acid hexahydrate (H_2_PtCl_6_·6H_2_O) which were both purchased from Sinopharm Chemical Reagent Co., Ltd. In addition, XC72, one of carbon supports adopted in the present study, was purchased from Shanghai Titan Technology Co. Multiwalled CNTs (30–50 nm in diameter and 30–100 μm in length) and monolayer graphene oxide (GO, 0.8–1.2 nm in thickness) were received from Jiangsu Xianfeng Nanomaterials Technology Co. Gases used in this study were all classified as high purity grade (N_2_, 99.999%, O_2_, 99.999%, Air Liquide). All raw materials were used as received without any further purification.

### 2.2 Preparation of carbon supports

Prior to supported Pt catalyst preparation, two series of carbon composite supports were formulated, including the GO-XC72 composite and the CNT-XC72 composite. PDDA was utilized to functionalize the multiwalled CNTs beforehand to modify the surface chemical properties of CNTs (Zhu et al., 2018). Briefly, 300 mg of CNTs were slowly added to a 1,200 mL aqueous solution containing 0.5 wt.% PDDA and 1 wt.% NaCl. The mixture was sonicated for 1 h and stirred for 12 h to obtain a well-mixed CNTs-containing suspension. Afterward, the suspension was filtered, and the filter cake was washed repeatedly with deionized water to ensure that the excess PDDA in the filter cake was removed. Finally, the filter cake was dried in a vacuum oven at 80°C for 24 h to obtain the PDDA-functionalized CNTs.

Four GO-XC72 composites were arranged in the present study depending on the weight of GO in each composite changing from 5% to 30% and named as Pt/5%GO-XC72, Pt/10%GO-XC72, Pt/20%GO-XC72, and Pt/30%GO-XC72, respectively. Similarly, four CNT-XC72 composites were lining-up by varying the weight of functionalized CNTs from 10% to 40% in each composite and named as Pt/10%CNT-XC72, Pt/20%CNT-XC72, Pt/30%CNT-XC72, and Pt/40%CNT-XC72, respectively. For making up of above carbon composites, a typical procedure was as follows: 0.6 g of powdery carbon mixture was dispersed in 30 mL EG solution and subjected to ultrasonic mixing for 1 h. The carbon composite-containing EG suspension was thus obtained ready for the next catalyst preparation step.

### 2.3 Preparation of catalysts

Pt nanoparticles were loaded onto different carbon supports (XC72, GO-XC72, CNT, and CNT-XC72) via EG reduction method (Nguyen et al., 2023) with a Pt loading of 40 wt%. H_2_PtCl_6_·6H_2_O was used as the Pt precursor. In a typical procedure, the carbon composite-containing EG suspension prepared in the previous step was transferred to a 250 mL three-necked flask placed in a magnetic stirring device. Under stirring at high speed along with Ar gas purging, 20 mL chloroplatinic acid-ethylene glycol precursor solution (0.0191 g_Pt_·mL^-1^) was added dropwise to the flask within *ca.* 20 min, and then the resultant suspension was continuously stirred overnight at room temperature. Afterwards, the pH of the suspension in the flask was adjusted to 13 with a solution of NaOH (2 mol L^-1^) in EG accompanied with a continuous stirring for 4 h. Following that, an oil bath was employed to heat the suspension from room temperature to 130°C within 30 min and the heating was maintained at 130°C for 4 h under stirring. The suspension was then allowed to cool down naturally to room temperature. This time, the pH of the suspension was adjusted to below 3 with an HCl solution (2 mol L^-1^). After standing for 10 min, the suspension was filtered using a vacuum pump, and the filter cake was washed several times with deionized water until the filtrate reaching the pH value of 6. The filter cake was finally dried in a vacuum oven at 80°C for 24 h. The dried sample was ground in an agate mortar and transferred to a sample tube for storage, ready for use as a Pt/C catalyst.

### 2.4 Electrochemical properties measurement

The electrochemical activity of catalyst was evaluated on a rotating disk electrode (RDE) utilizing a CHI760 electrochemical workstation. The catalyst ink was prepared by dispersing 5 mg of Pt/C catalyst in a solution containing Nafion ionomer composed of 500 μL ultrapure water, 500 μL isopropanol, and 25 μL 5 wt.% Nafion. Then 10 μL catalyst ink was coated on a glassy carbon electrode (5 mm in diameter, Pine Instruments), and the electrode was attached to the shaft of the electrode rotator (Pine, AFMSRX). A saturated calomel electrode (SCE) was used as a reference electrode. A platinum wire electrode was employed as counter electrode. All the RDE tests were conducted at 25 °C in a 0.1 mol L^-1^ HClO_4_ electrolyte. Cyclic voltammetry (CV) scan was performed in an Ar-saturated HClO_4_ electrolyte by circulating the electrical potential from 0.25 to 1.2 V vs. RHE at different scanning rates (10–1,000 mV s^-1^) to acquire the electrochemically active surface area (ECSA) of each Pt/C catalyst and the accessibility of Pt nanoparticles on/in carbon support. The background current was also measured in the Ar-saturated electrolytic cell. For the measurement of catalyst performance during ORR process, linear sweep voltammetry (LSV) was carried out using O_2_-saturated HClO_4_ electrolyte in the electrical potential range from 0.375 to 1.05 V (vs. RHE) with a scanning rate of 10 mV s^-1^ and an adjustable rotation rate among 400–2,500 rpm. To conduct accelerated durability test (ADT), the catalyst electrode in the O_2_-saturated electrolytic cell is scanned using CV method in the range of 0.6–1.0 V (vs. RHE) at a rate of 100 mV s^-1^ for 5,000 and 10,000 cycles. The CV and LSV curves were recorded after a certain number of cycles, as described above, to evaluate the durability of the catalyst tested.

The *ECSA* (m^2^·g_Pt_^-1^) of a Pt/C catalyst was calculated from the area of the hydrogen adsorption peaks in the corresponding CV curve following the equation below:

$$ECSA=\frac{Q_{H_{2}}}{C\cdot v\cdot m_{Pt}}$$ (1)

where $Q_{H_{2}}$ (C·m^-2^) is the amount of electricity derived from the integrated area of the hydrogen desorption peak; *v* (mV·s^-1^) is the scanning rate of the cyclic voltammetry; C (C·m^-2^) is the theoretical adsorption power of hydrogen atoms in a smooth Pt crystal monolayer and here we took 2.1 C m^-2^; *m*_Pt_ (g) is the Pt loading amount on the electrode.

The mass activity (*MA*, A·g^-1^) was calculated from the following equation:

$$j_{k}=\frac{j_{0.9\cdot }j_{\lim}}{j_{0.9}{-j}_{\lim}}$$ (2)

$$MA=\frac{j_{k}}{m_{Pt}}$$ (3)

where *j*_k_ (A·m^-2^) is the kinetic current density; *j*_lim_ (A·m^-2^) is the measured diffusion-limiting current density, and *j*_0.9_ (A·m^-2^) is the current density at 0.9 V (vs. RHE).

### 2.5 Characterization of catalysts

The textural properties of various Pt/C catalysts, including specific surface area, pore volume, and pore size distribution, were measured using an automatic N_2_ cryogenic physical adsorption instrument followed by Brunauer-Emmett-Teller (BET) and Barrett-Joyner-Halenda (BJH) methodologies (Micromeritics ASAP 2020). The porosity of a Pt/C catalyst is derived by the following equation (Suzuki et al., 2011):

$$\varepsilon =\frac{V_{p}}{V_{p}+\frac{m_{nafion}}{\rho_{nafion}\cdot m_{carbon}}+\frac{m_{Pt}}{\rho_{Pt}\cdot m_{carbon}}+\frac{1}{\rho_{carbon}}}$$ (4)

where $V_{p}$ (m^3^·g^-1^) is the total pore volume based on N_2_ cryogenic physical adsorption approach; $m_{nafion}$ , $m_{carbon}$ and $m_{Pt}$ (g) are the mass of Nafion, carbon support and Pt, respectively. $\rho_{nafion}$ , $\rho_{carbon}$ and $\rho_{Pt}$ (g·m^-3^), the density of corresponding material, are 2.1 × 10^6^ g m^-3^, 2 × 10^6^ g m^-3^ and 2.145 × 10^7^ g m^-3^, respectively, as we take here.

Pt loading was determined by the inductively coupled plasma mass spectrometry (ICP-MS, Perkinelmer Co., Ltd.). The catalyst microstructure and the size of Pt nanoparticles were detected using a high-resolution transmission electron microscopy (HR-TEM, JOEL JEM-2100), and the catalyst morphology was observed on a field emission scanning electron microscopy (SEM, Nova NanoSEM 450). X-ray diffraction detection (XRD) method was applied to characterize the crystal phase and Pt particle size of the catalysts (D8 Advance Da Vinci type), which was fulfilled by adopting copper target radiation (*λ* = 0.15405 nm) along with an operative voltage of 40 kV and a current of 40 mA. The scanning range was 2*θ* = 5–90°and the scanning rate was *ω* = 1°min^-1^. The Scherrer formula was used to compute the size of Pt nanoparticles.

### 2.6 Oxygen transport resistance calculation

**FIGURE 1:**
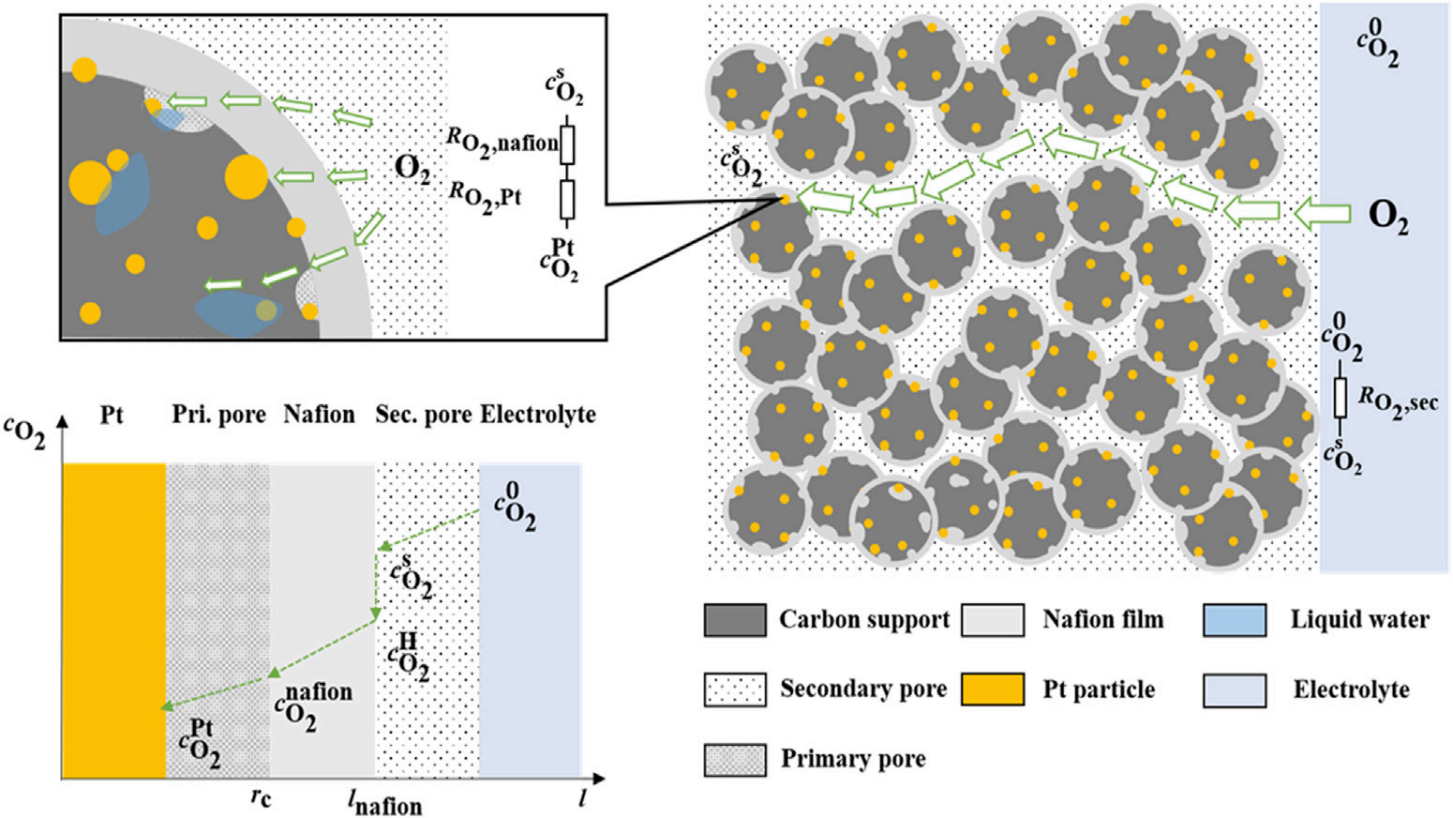
Schematic diagram of oxygen transport through catalyst layer of electrode.

Figure 1 illustrates the schematic diagram of the oxygen transfer route in the electrode. The total diffusion resistances (*R*_total_, s·m^-1^) of oxygen from the electrolyte to the reaction site are governed by two mass transfer resistances: one in the secondary pore and one at the local site. The oxygen local transport resistance (*R*_local_, s·m^-1^) can be further partitioned into two distinct components: $R_{O_{2},nafion}$ (s·m^-1^), which arises due to the transport of oxygen through the ionomer film, and $R_{O_{2},Pt}$ (s·m^-1^), which is caused by carbon agglomeration and Pt particle distribution and Knudsen diffusion (Xie et al., 2019). $c_{O_{2}}^{0}$ is the concentration of dissolved oxygen in the electrolyte; $c_{O_{2}}^{s}$ is the oxygen concentration after passing through the secondary bore; $c_{O_{2}}^{H}$ (mol·m^-3^) is the oxygen concentration to reach the surface of the Nafion film; $c_{O_{2}}^{nafion}$ (mol·m^-3^) is the oxygen concentration after passing through the Nafion film; $c_{O_{2}}^{Pt}$ (mol·m^-3^) is the oxygen concentration reaching the Pt particle:

$$R_{total}=R_{O_{2},\mathrm{Sec}}+R_{local}$$ (5)

$$R_{local}=R_{O_{2},nafion}+R_{O_{2},Pt}$$ (6)

A homogeneous and stable surface diffusion can be established on the rotating disc electrode, where the reaction current and the limiting diffusion current satisfy the Koutecky-Levich (K-L) relationship under strongly polarized conditions (Masa et al., 2014):

$$\frac{1}{j}=\frac{1}{j_{k,app}}+\frac{1}{j_{nafion}}+\frac{1}{j_{\lim}}$$ (7)

where $j_{nafion}$ (A·m^-2^) is the diffusion current corresponding to the diffusive mass transfer of oxygen in the Nafion ionomer; $j_{\lim}$ (A·m^-2^) is the limit current for diffusion control; $j_{k,app}$ (A·m^-2^) is the apparent kinetic current of the electrode, which is considered to be determined jointly by the intrinsic kinetic current $j_{k,ture}$ and the kinetic current affected by internal diffusion $j_{k,\mathrm{int}}$ :

$$\frac{1}{j_{k,app}}=\frac{1}{j_{k,ture}}+\frac{1}{j_{k,\mathrm{int}}}$$ (8)

When the kinetics of the electrode is in electrochemical step control, the relationship between the overpotential and current density of the electrode kinetic process can be given by the Bulter-Volmer equation, and the apparent kinetic current can hence be expressed as:

$$j_{k,app}=\frac{c_{O_{2}}^{Pt}}{c_{O_{2}}^{ref}}j^{ref}{\cdot k}_{T}\left(1-{k_{Pt/O}}_{2}\right)\exp\left(-\frac{\alpha_{C}\cdot F\cdot \theta }{RT}\right)$$ (9)

$$k_{T}=\exp\left(-\frac{7900\left(353.15-T\right)}{353.15\cdot T}\right)$$ (10)

$$k_{Pt/O_{2}}=\frac{1}{1+e^{22.4\left(0.818-V\right)}}$$ (11)

where $c_{O_{2}}^{ref}$ (mol·m^-3^) is the reference oxygen concentration, taken as 3.39 mol m^-3^ (Xie et al., 2019) here, and $j^{ref}$ (A·m^-2^) is the reference current density, taken as 1.9 A m^-2^. $k_{T}$ is the temperature correction coefficient, and $k_{Pt/O_{2}}$ is the Pt/O_2_ coverage dependence correction coefficient; $\alpha_{C}$ is the cathodic transfer coefficient, taken as 0.5; F (C·mol^-1^) is the Faraday’s constant; θ (V) is the overpotential.

The mass transfer resistance in the secondary pore can be expressed as:

$$R_{O_{2},\mathrm{Sec}}=\frac{l_{CL}}{D_{O_{2},\mathrm{Sec}}\cdot \varepsilon^{1.5}}$$ (12)

$l_{CL}$ (m) is the thickness of catalyst layer; $D_{O_{2},\mathrm{Sec}}$ (m^2^·s^-1^) is the diffusion coefficient in the secondary pores, taken as 1.426 × 10^−5^ (Xie et al., 2019) here; ε is the porosity of catalyst.

The flux of oxygen diffusion through secondary pore can be expressed as (Xie et al., 2019):

$$N_{O_{2},\mathrm{Sec}}=\frac{j}{4F}\frac{A_{Pt}}{A_{nafion}}=\frac{c_{O_{2}}^{0}-c_{O_{2}}^{s}}{R_{O_{2},\mathrm{Sec}}}$$ (13)

$$A_{Pt}=\frac{m_{Pt}\cdot ECSA}{l_{CL}}$$ (14)

$$A_{nafion}=A_{Pt}\left(1+\frac{l_{nafion}}{r_{carbon}}\right)$$ (15)

$A_{Pt}$ (m^2^·m^-3^) is the specific surface area of Pt particle in the catalyst layer; $m_{Pt}$ (kg·m^-2^) is the specific Pt loading; $A_{nafion}$ (m^2^·m^-3^) is the specific surface area of nafion film; $l_{nafion}$ (m) is the thickness of nafion film; $r_{carbon}$ (m) is the radius of carbon support.

The flux of oxygen dissolved by diffusion from secondary pores through Nafion can be expressed as:

$$N_{O_{2},nafion}=\frac{A_{Pt}}{A_{nafion}}\frac{j_{nafion}}{4F}=\frac{c_{O_{2}}^{H}-c_{O_{2}}^{nafion}}{R_{O_{2},nafion}}$$ (16)

$$c_{O_{2}}^{H}=\frac{c_{O_{2}}^{s}RT}{H_{O_{2}}}$$ (17)

$$H_{O_{2}}=0.101325\exp\left(14.1-\frac{666}{T}\right)$$ (18)

$$R_{O_{2},nafion}=k_{Pt/nafion}\frac{l_{nafion}}{D_{O_{2},nafion}}$$ (19)

$H_{O_{2}}$ (Pa·m^3^·mol^-1^) is the Henry factor for oxygen in ionomers; R (J·mol^-1^·K^−1^) is the universal gas constant; *T* (K) is the local temperature; $R_{O_{2},nafion}$ (s·m^-1^) is the oxygen transfer resistance across the Nafion film. Since the catalyst surface is not completely covered by the Nafion film, a correction factor $k_{Pt/nafion}$ is introduced. $D_{O_{2},nafion}$ (m^2^·s^-1^) is the oxygen diffusivity in ionomer, calculated as follows, where λ is the aqueous molecular weight on the sulfonic acid groups of the ionomer (Xing et al., 2014):

$$D_{O_{2},nafion}=1.392\times 10^{-10}\lambda^{0.708}\exp\left(\frac{T-273.15}{106.65}\right)-1.646\times 10^{-10}\lambda^{0.708}+5.2\times 10^{-10}$$ (20)

The diffusion current corresponding to the diffusive mass transfer of oxygen in a Nafion film can again be expressed as:

$$j_{nafion}=\frac{4F\left(c_{O_{2}}^{H}-c_{O_{2}}^{nafion}\right)}{\frac{A_{Pt}}{A_{nafion}}R_{O_{2},nafion}}$$ (21)

The flux of oxygen diffusing from the Nafion to the Pt surface is given as:

$$N_{O_{2},Pt}=\frac{j_{k,app}}{4F}=\frac{c_{O_{2}}^{nafion}-c_{O_{2}}^{Pt}}{R_{O_{2},Pt}}$$ (22)

The calculation of $R_{O_{2},Pt}$ is complicated, because the diffusion resistance in water and in ionomer, as well as Knudsen resistance, need to be taken into account (Xie et al., 2019):

$$R_{O_{2},Pt}=\frac{l_{Pt}^{eq}}{D_{O_{2},Pt}^{eq}}=l_{Pt}^{eq}\left(\frac{1}{D_{O_{2},lw}}+\frac{1}{D_{O_{2},kn}}\right)$$ (23)

The gas model may be used to obtain the Knudsen diffusivity as (Mench, 2008; Lange et al., 2010):

$$D_{O_{2},kn}=\frac{8r_{pore}}{3}\sqrt{\frac{RT}{2\pi M}}$$ (24)

$l_{Pt}^{eq}$ (m) is the equivalent effective length related to $r_{carbon}$ ; $D_{O_{2},lw}$ (m^2^·s^-1^) is the oxygen diffusivity in liquid water (Chen et al., 2018), and $D_{O_{2},kn}$ (m^2^·s^-1^) is the Knudsen diffusion coefficient; M (kg·mol^-1^) is the molecular weight of oxygen; $r_{pore}$ (m) is the pore radius that tested by BET.

Combining (7) and (18) and eliminating the $c_{O_{2}}^{Pt}$ term give the apparent current density expression as:

$$j_{k,app}=\frac{4F\cdot c_{O_{2}}^{nafion}}{\frac{4F\cdot c_{O_{2}}^{ref}}{k_{ele}}+\frac{A_{Pt}}{A_{nafion}}R_{O_{2},Pt}}$$ (25)

$$k_{ele}=j^{ref}k_{T}\left(1-{k_{Pt/O}}_{2}\right)\exp\left(-\frac{\alpha_{C}F\theta }{RT}\right)$$ (26)

Revising this to an expression that conforms to the K-L relationship:

$$\frac{1}{j_{k,app}}=\frac{1}{\frac{c_{O_{2}}^{nafion}}{c_{O_{2}}^{ref}}k_{ele}}+\frac{1}{\frac{c_{O_{2}}^{nafion}}{\frac{A_{Pt}}{A_{nafion}}R_{O_{2},Pt}}4F}$$ (27)

An expression for the intrinsic current and the internal diffusion resistance term can be obtained as:

$$j_{k,true}=\frac{c_{O_{2}}^{nafion}}{c_{O_{2}}^{ref}}k_{ele}$$ (28)

$$j_{k,\mathrm{int}}=\frac{c_{O_{2}}^{nafion}}{\frac{A_{Pt}}{A_{nafion}}R_{O_{2},Pt}}4F$$ (29)

$$\frac{c_{O_{2}}^{ref}}{c_{O_{2}}^{nafion}k_{ele}}+\frac{\frac{A_{Pt}}{A_{nafion}}R_{O_{2},Pt}}{4Fc_{O_{2}}^{nafion}}+\frac{\frac{A_{Pt}}{A_{nafion}}R_{O_{2},Pt}}{4F\left(c_{O_{2}}^{H}-c_{O_{2}}^{nafion}\right)}=\frac{1}{j_{k,app}}+\frac{1}{j_{nafion}}=\omega$$ (30)

where ω is the reciprocal of the kinetic current measured by the rotating disc electrode.

Based on the definition of the internal diffusion efficiency factor, *η* can thus be obtained for the purposes of this study as:

$$\eta =\frac{c_{O_{2}}^{Pt}}{c_{O_{2}}^{0}}$$ (31)

The value of $c_{O_{2}}^{Pt}$ can be solved with the aid of Matlab software.

## 3 Results and discussion

### 3.1 Textural feature of Pt catalysts supported on different carbon materials

**FIGURE 2:**
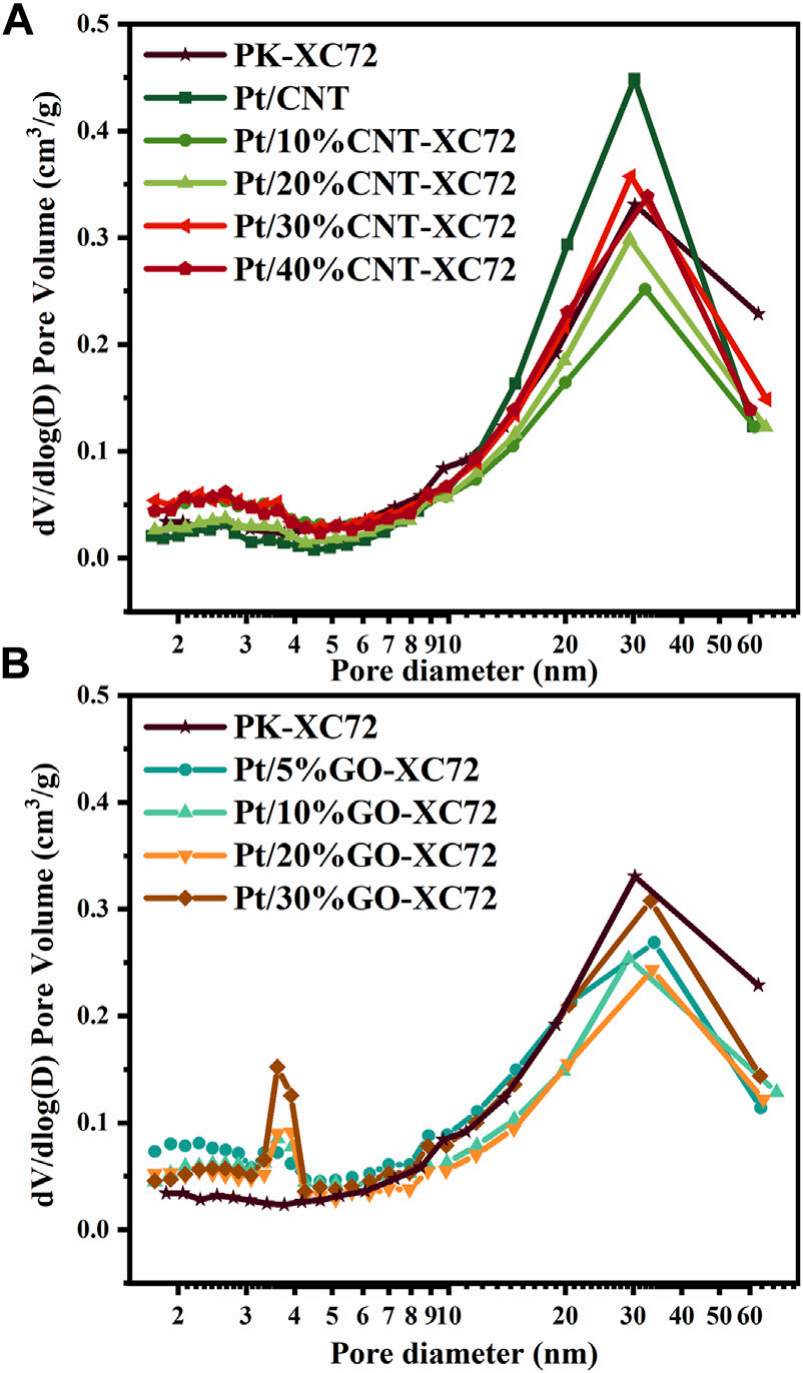
Pore size distribution of various Pt/C catalysts. **(A)** Pt/CNT-XC72 series; **(B)** Pt/GO-XC72 series.

The textural properties of different Pt/C catalysts were characterized using N_2_ physisorption method. Figure 2 shows the pore size distribution of two series of catalysts with different carbon supports. In the present study, we categorized the pore distribution into two distinct parts: the surface primary pores (2–10 nm) and the secondary pores (10–100 nm). The primary pores were actually caused by surface sags and crests of the carbon particles because of solid nature of both XC72 and GO and as well as inaccessible CNT lumen; all of these carbon materials lack accessible internal pores, unlike Ketjen Black, which is rich in inner pores (Yarlagadda et al., 2018). The agglomeration of carbon particles constituted the secondary pores. Accordingly, the two types of pore structure should take specific responsibility for the oxygen diffusion through the layer of a Pt/C catalyst. The Pt loading, specific surface area, pore volume, and porosity of each catalyst are summarized in Table 1.

**TABLE 1:** Specific surface area, pore volume and porosity of Pt catalysts with different carbon supports.

Sample	Pt loading (wt.%)	Specific surface area (m^2^·g^-1^)	Pore volume (cm^3^·g^-1^)			Porosity (%)
			Primary pores (2–10 nm)	Secondary pores (10–100 nm)	Total volume	
PK-XC72	40	72.408	0.028	0.345	0.373	33.08
Pt/CNT	35	58.282	0.020	0.287	0.307	28.92
Pt/10%CNT-XC72	45	78.044	0.038	0.257	0.295	28.10
Pt/20%CNT-XC72	46	70.635	0.035	0.217	0.252	25.03
Pt/30%CNT-XC72	42	107.781	0.036	0.211	0.247	24.66
Pt/40%CNT-XC72	44	86.804	0.036	0.264	0.300	28.44
Pt/5%GO-XC72	42	148.019	0.054	0.228	0.283	27.27
Pt/10%GO-XC72	40	96.273	0.045	0.203	0.248	24.73
Pt/20%GO-XC72	37	104.972	0.039	0.199	0.239	24.05
Pt/30%GO-XC72	43	99.913	0.049	0.256	0.306	28.85

The influence of CNT addition on the textural properties of the Pt/CNT-XC72 series catalysts can be appraised from the curves in Figure 2A and the data in Table 1. Although the CNTs have an inferior specific surface area, mixing CNTs of more than 30% with XC72 can coarsen the material surface by extending the surface roughness, which are probably formed through electrostatic attraction between the interfaces of CNTs and XC72 particles (Shi et al., 2017). In contrast to the faint primary pore peaks, the secondary pores of all the Pt/CNT-XC72 catalysts cresting around 30 nm are conspicuous. The presence of CNTs in the carbon supports decreases the secondary pore volume in comparison with PK-XC72 which uses single XC72 material as the Pt support. A minimum secondary pore volume appears when the weight of CNTs in the carbon composite approaches 30%. A similar trend is valid for the change in the porosity, indicating the dominant contribution of secondary pores to the total porosity.

Figure 2B presents the pore size distribution of the Pt/GO-XC72 series catalysts. Two peaks of pore size can be clearly distinguished; one is centered on 3.8 nm, and another is around 30 nm. The former can be assigned to the primary pores, probably associated with the fold of GO flakes, while the latter can be attributed to the secondary pores circumscribed by the aggregation of carbon particles. The addition of GO into XC72 for the formation of composite supports significantly increases the primary pore volume of the supported Pt catalysts compared to the reference PK-XC72 catalyst using XC72 support. Furthermore, the specific surface area of the catalysts is also boosted after adding a variable quantity of GO. Nevertheless, the volume of secondary pores changing with the amount of GO is complicated. Similar to the Pt/CNT-XC72 series catalysts, the Pt/GO-XC72 series catalysts also have the lowest secondary pore volume and porosity, both presented by the Pt/20%GO-XC72 sample. Therefore, the mixing of XC72 with CNT or GO can effectively modify the texture of the components, making the amelioration of the internal diffusion problem for the catalysts possible.

**FIGURE 3:**
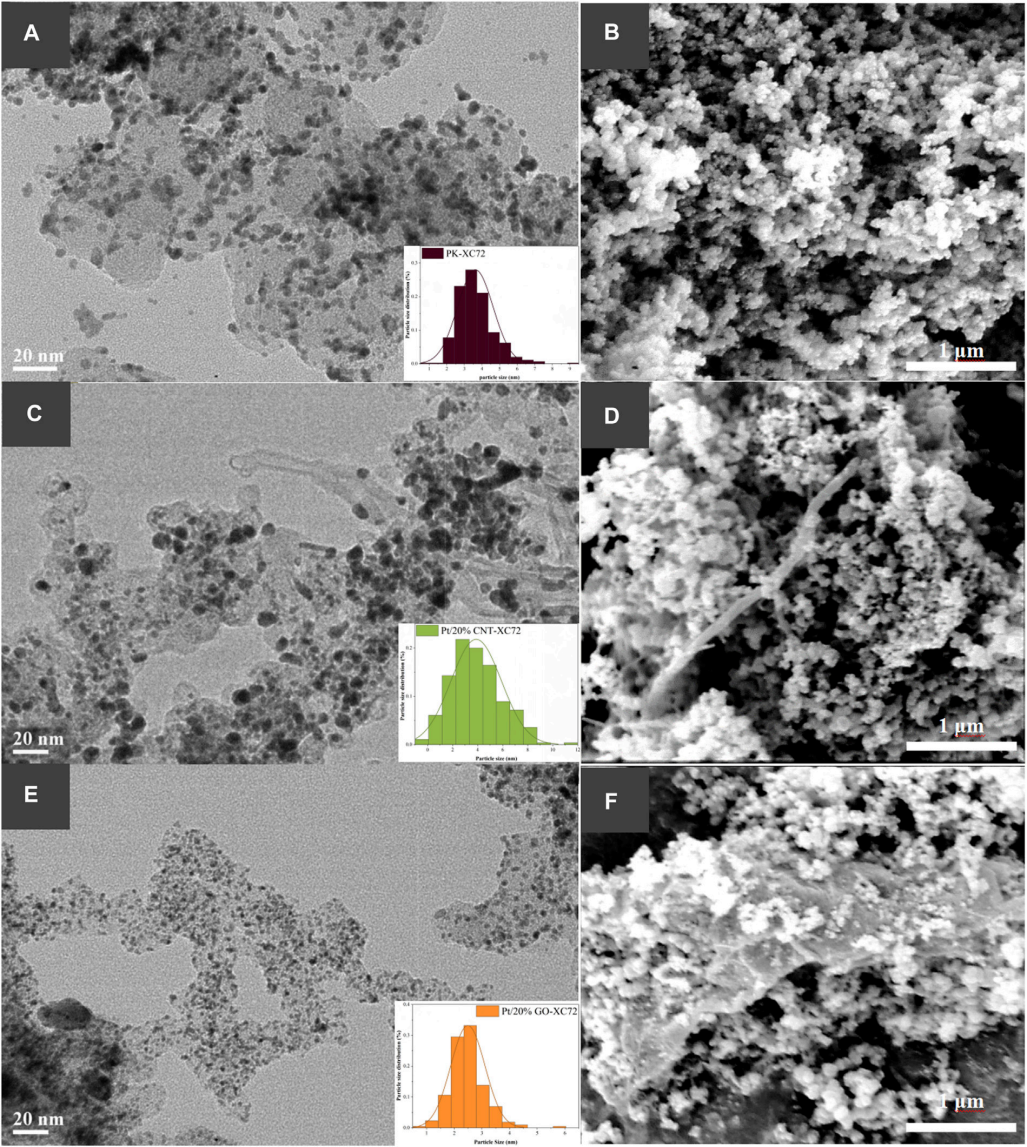
HR-TEM and SEM images showing Pt nanoparticle distribution and morphology of Pt catalysts on different carbon supports. **(A, B)**: PK-XC72; **(C, D)**: Pt/20%CNT-XC72; **(E, F)**: Pt/20%GO-XC72.

The morphology and the Pt nanoparticles dispersion state of the representative Pt/20%CNT-XC72 and Pt/20%GO-XC72 catalysts, as well as the reference PK-XC72 catalyst, are illustrated as HR-TEM images in Figure 3. More HR-TEM images of Pt/CNT-XC72 series and Pt/GO-XC72 series catalysts are shown in Supplementary Figure S1 of Supplementary Materials. It can be observed from Figures 3A–D that the overall morphology of the catalyst changes considerably with the addition of CNTs. The high aspect ratio of CNTs gives the catalyst a looser and more porous structure, which may facilitate gas transport through the catalyst layer. While due to the extensive flake structure of GO compared to carbon black agglomerates, it seems no distinct gas transport channels formed after adding GO into XC72, as shown in Figures 3E,F, which need further investigation.

Moreover, the Pt nanoparticles are uniformly distributed on the composites and simple carbon black support. Some of the Pt nanoparticles distributed on the 20%CNT-XC72 support (Figure 3C) are larger compared to those on XC72 support (Figure 3A). In contrast, the Pt nanoparticles on the 20%GO-XC72 support seem much smaller (Figure 3E). Undoubtedly, the surface properties of CNTs and GO can bring about a different distribution of Pt nanoparticles.

**FIGURE 4:**
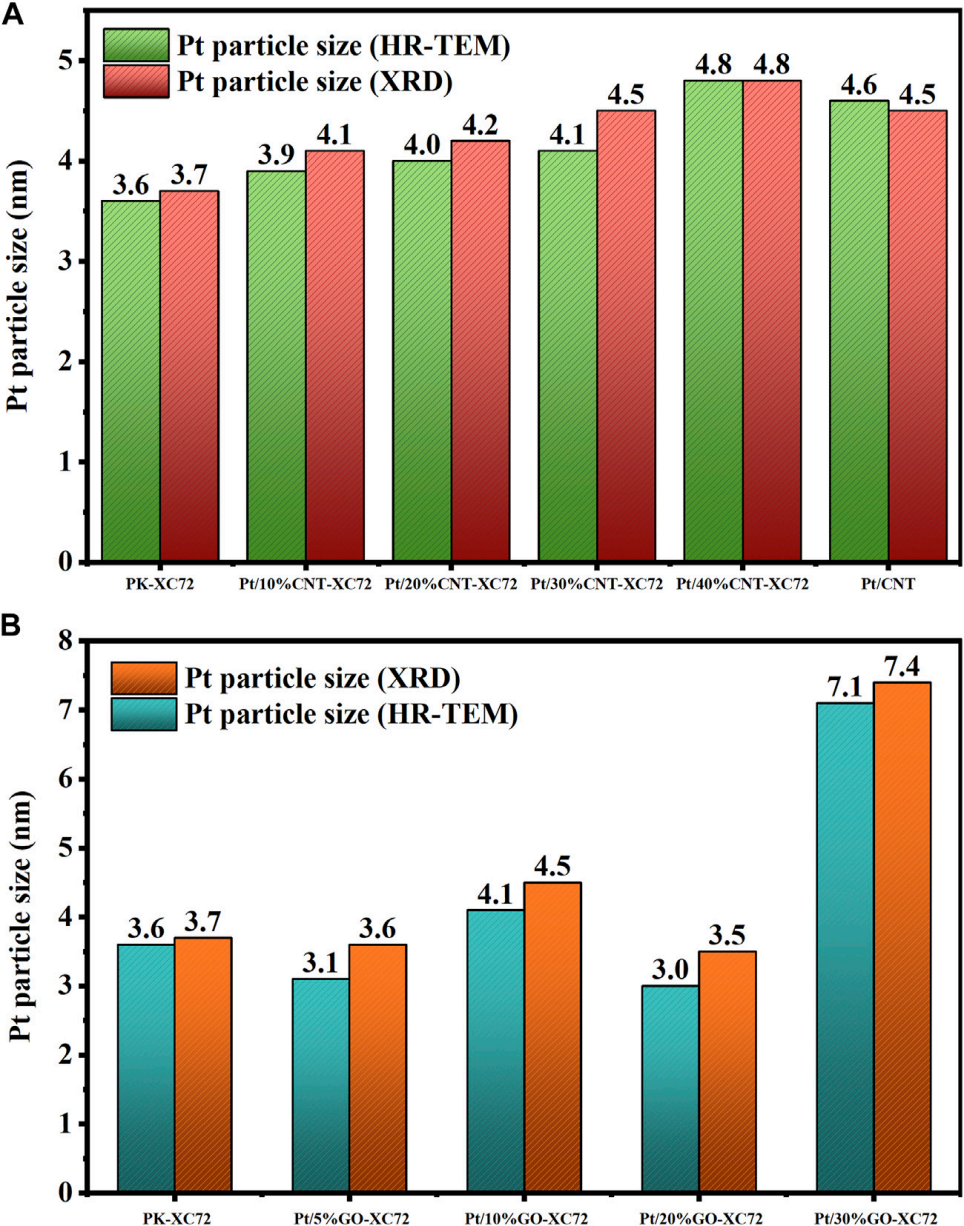
Pt particle size measured by HR-TEM and XRD methods. **(A)** Pt/CNT-XC72 series; **(B)** Pt/GO-XC72 series.

The size of the Pt nanoparticles on every carbon material involved in the present study was quantified by both HR-TEM and XRD approaches and is compared in Figure 4. The XRD spectra of two series of catalysts are supplied in Supplementary Figure S2 of Supplementary Materials. The results of XRD derived using the Scherrer formula and those of HR-TEM from particle statistics are reasonably consistent with each other. As the addition of CNTs increases, the average particle size of the Pt nanoparticles also increases, indicating a negative effect of CNTs on Pt nanoparticles dispersion. In contrast, the addition of GO below 30% does not significantly change the size of Pt nanoparticles. But for the sample of Pt/30%GO-XC72, the average Pt particle size exceeds 7 nm, likely arising from the uneven distribution of Pt nanoparticles on poor mixed XC72 with a large quantity of GO.

### 3.2 Electrochemical properties

**FIGURE 5:**
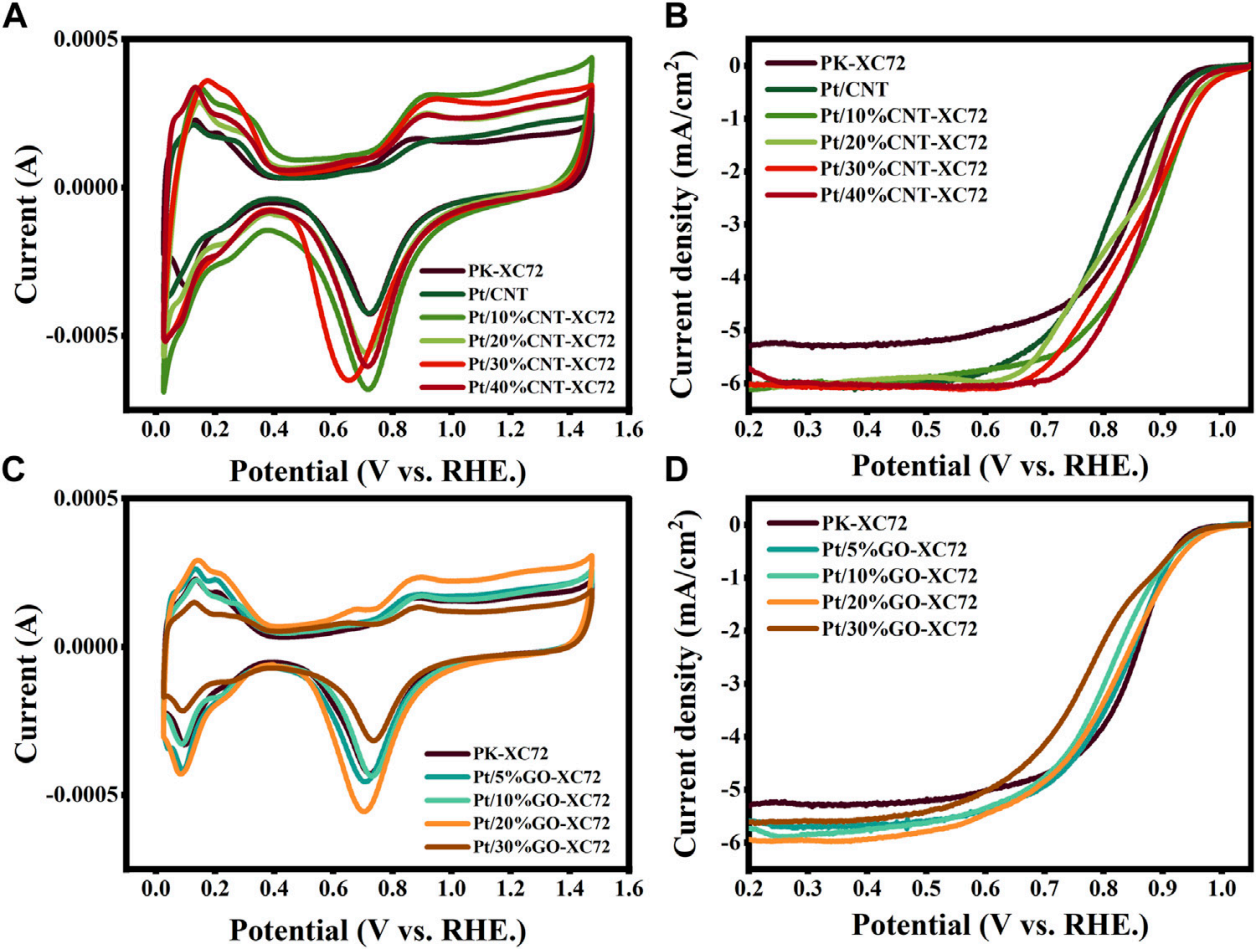
CV **(A)** and LSV **(B)** curves of Pt/CNT-XC72 series; CV **(C)** and LSV **(D)** curves of Pt/GO-XC72 series.

**FIGURE 6:**
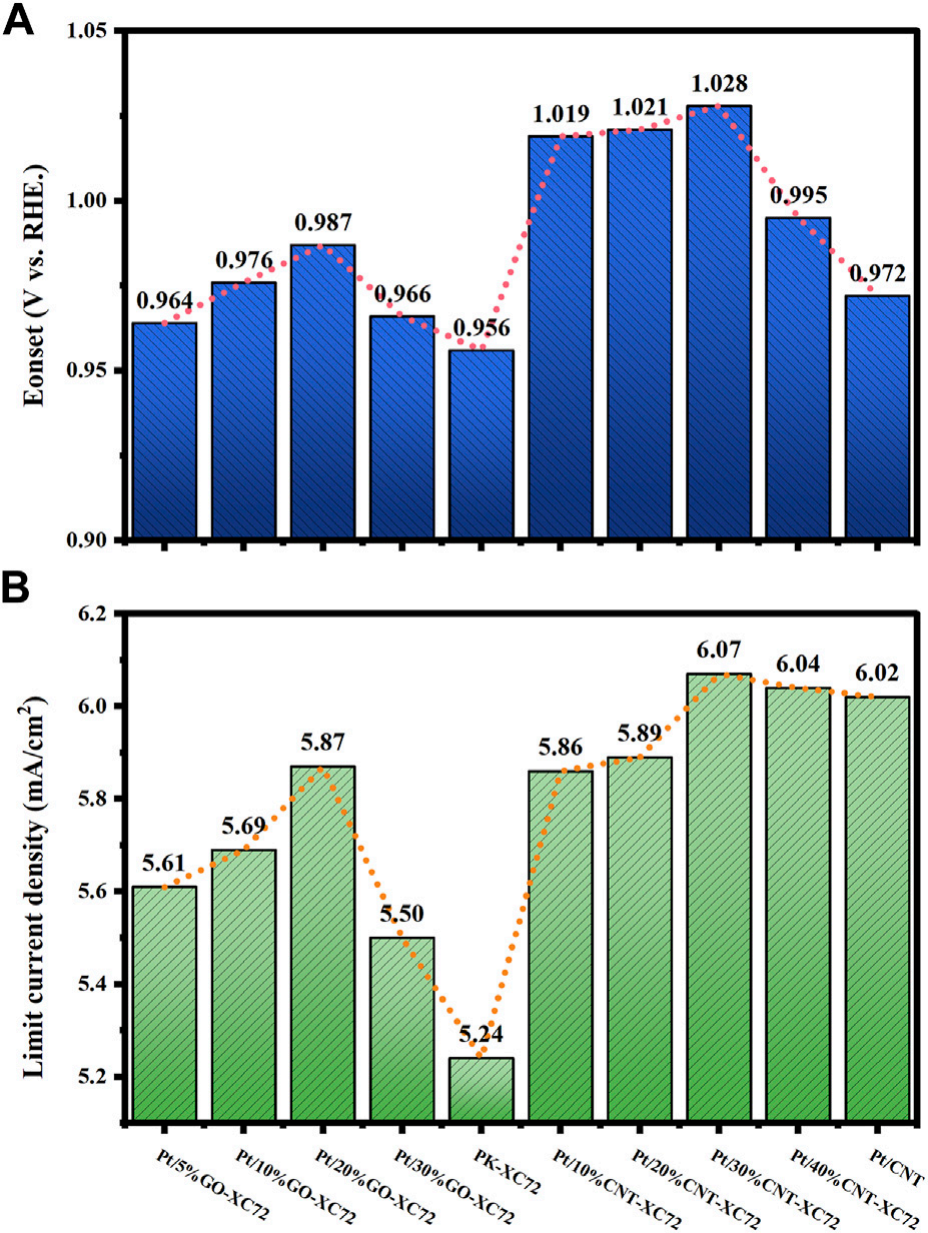
Electrochemical properties of the catalysts. **(A)** *E*_onset_; **(B)** limiting current density.

The ORR activity of the catalysts was measured using CV method in Ar and O_2_ saturated 0.1 M HClO_4_ solution by rotating a disc electrode with scan rate of 10 mV s^-1^, 20 mV s^-1^, 50 mV s^-1^, 100 mV s^-1^, 200 mV s^-1^, 400 mV s^-1^, 600 mV s^-1^, 800 mV s^-1^, 1,000 mV s^-1^, respectively. LSV curve was recorded at a scan rate of 10 mV s^-1^ and 1,600 rpm, before which 20 cycles of potential cycling were conducted so as to obtain stable profiles and net background currents. Figure 5 shows the results of the CV and LSV test for the different catalysts. The limiting diffusion current density was gained from the LSV curve at 0.4 V (vs. RHE). The onset potential (*E*_onset_) was defined as the potential at which the current density reached 0.1 mA cm^-2^. The results for all the samples are collected and drawn with histogram in Figure 6.

It can be seen that in comparison with the commercial PK-XC72 catalyst, both the onset potential and the limiting diffusion current density are increased when selecting the hybrid carbon black supports, indicating an increase in ORR activity after adding CNT or GO into XC72. In particular, the Pt/30%CNT-XC72 and Pt/20%GO-XC72 samples have an onset potential of 1.028 V and 0.987 V, respectively, which are significantly promoted as compared to the commercial catalyst of 0.956 V. The limiting diffusion current density of the composite-supported catalysts also performs better. The improved performance should be attributed to the addition of appropriate quantity of CNTs or GO, which gives the catalyst layer a more porous and regular structure, thus facilitating the localized mass transfer of oxygen and consequently fully utilizing the catalytic activity.

In research by Ott et al. (2020), it has been demonstrated that at high electrode scan rate, the mass transfer velocity at the catalyst surface fails to keep up with the reaction rate, resulting in a reduction in ECSA. Therefore, the active Pt sites on the carbon surface that is more accessible to the reactants can provide higher mass transfer efficiency, leading to a relatively gentle reduction in ECSA (Peng et al., 2021). In the present study, ECSA was measured at different scan rates for the evaluation of the accessibility of Pt nanoparticles.

**FIGURE 7:**
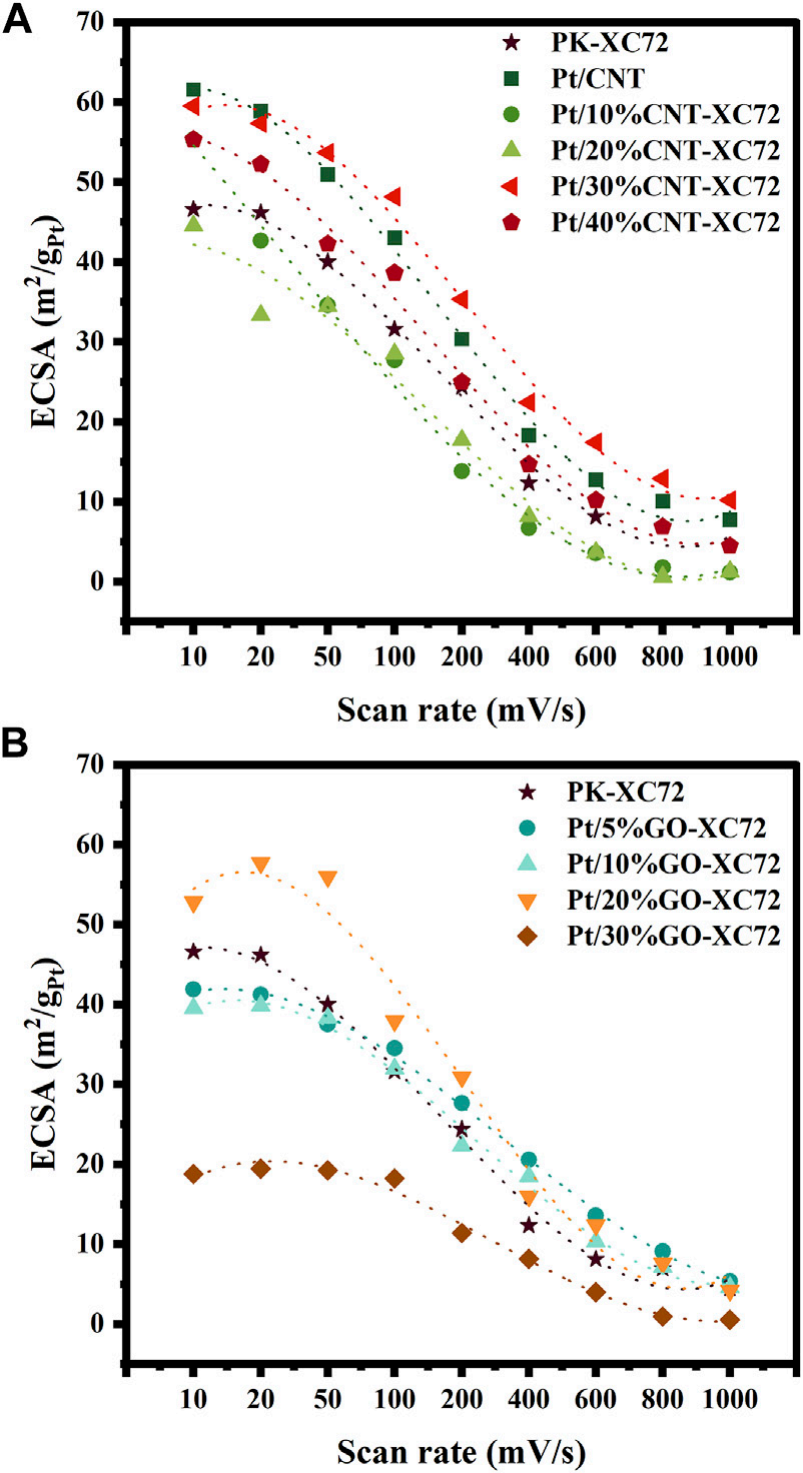
ECSA changing with scan rate and fitting curves. **(A)** Pt/CNT-XC72 series; **(B)** Pt/GO-XC72 series.

As shown in Figure 7A, at a low scan rate of 10 mV s^-1^, the Pt/CNT catalyst presents the highest ECSA value despite that it owns a considerably large Pt particle size, while the PK-XC72 provides a moderate ECSA although it possesses the smallest Pt nanoparticles. Mixing XC72 with a small amount of CNTs renders a slight decrease in ECSA if compared to PK-XC72, but a 30% or 40% addition of CNTs instead gives a higher ECSA than PK-XC72. Nevertheless, an increase in the scan rate significantly depresses the ECSA of all the samples. Specifically, raising the scan rate faster than 50 mV s^-1^, the decline in ECSA of Pt/30%CNT-XC72 was fairly slower than that of Pt/CNT catalyst, thereby affording the highest ECSA. This implies that the addition of 30%CNT into XC72 can enhance the accessibility of Pt nanoparticles on the carbon materials to some extent even if in comparison with Pt/CNT catalyst.

Figure 7B exhibits that the largest ECSA is achieved on the catalyst of Pt/20%GO-XC72 at a slower scan rate than 50 mV s^-1^. Furthermore, there is a relatively more extended plateau in the decrease curve of ECSA after the addition of GO compared to PK-XC72, implying a stronger tolerance of mass transfer hindrance for the catalysts having GO addition. In the condition of high scan rate, the GO-XC72 composites containing a small amount of GO still perform well. The overall results of ECSA changing with scan rate suggest that an appropriate amount of GO or CNT addition can substantially improve the accessibility of the Pt nanoparticles.

**FIGURE 8:**
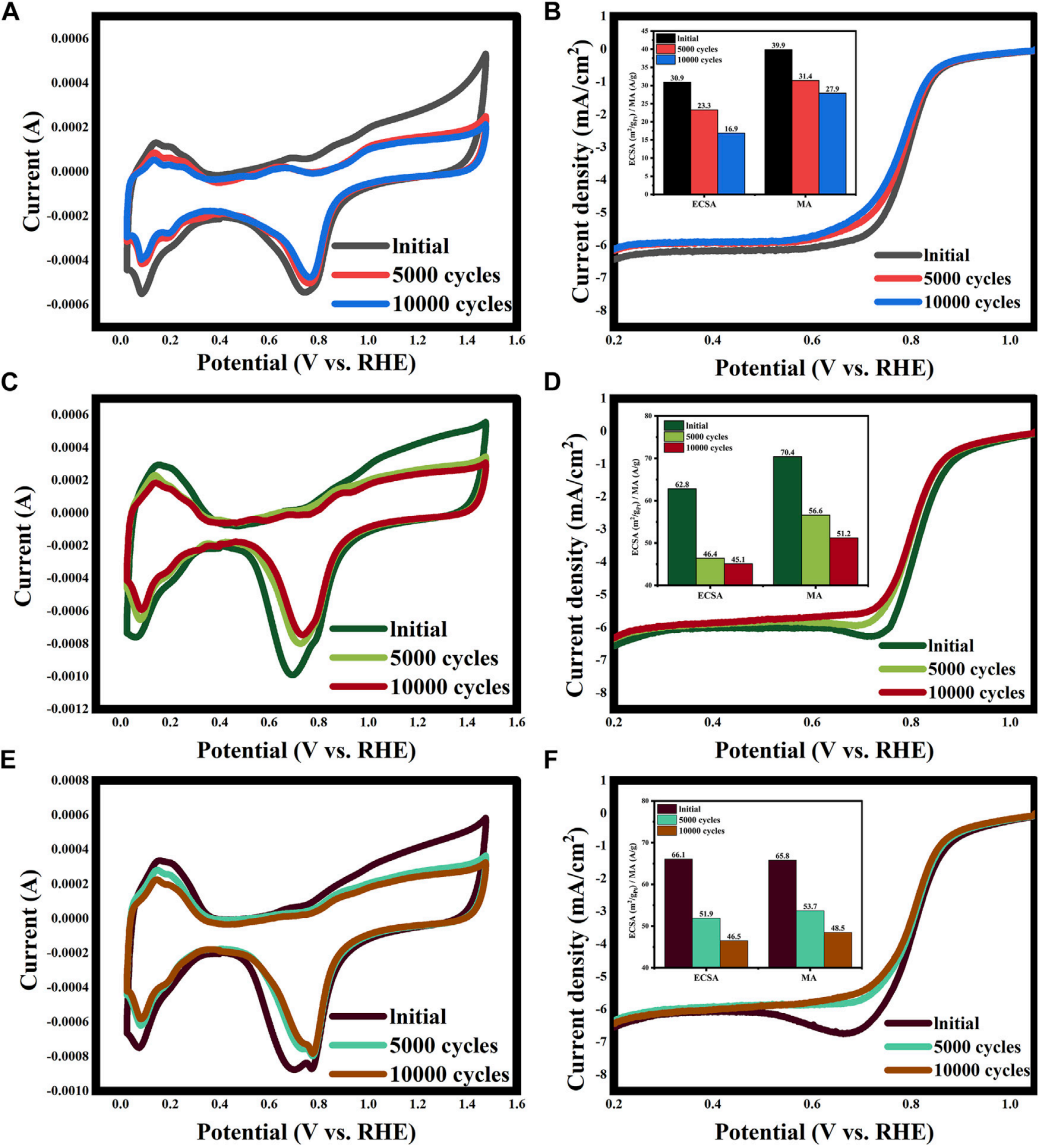
ADT test results of PK-XC72 **(A and B)**, Pt/30%CNT-XC72 **(C and D)**, and Pt/20%GO-XC72 **(E and F)**.

The ADT results of the Pt/30%CNT-XC72 and Pt/20%GO-XC72 compared with the commercial PK-XC72 catalyst are shown in Figure 8. It can be observed that after 5,000 and 10,000 cycles of accelerated aging, the catalysts show different degrees of performance degradation. The initial values of ECSA and MA for the commercial catalyst (Figure 8A and (B)) are 30.9 m^2^·g_Pt_^-1^ and 39.9 A g^-1^, respectively, which decrease by 24.6% and 21.3% after 5,000 cycles and by 45.3% and 30.1% after 10,000 cycles of accelerated aging, respectively. In contrast, the initial ECSA and MA of the Pt/30%CNT-XC72 catalyst (Figure 8C, D) are 62.8 m^2^·g_Pt_^-1^ and 70.4 A g^-1^, respectively, which fall by 26.1% and 19.6% after 5,000 cycles aging and by 28.2% and 27.3% after 10,000 cycles aging, respectively. Therefore, the ECSA and MA of the Pt/30%CNT-XC72 after 10,000 cycles aging are 2.7 times and 1.8 times those of the commercial catalyst, even higher than the initial values of the commercial catalyst. Clearly, the catalyst of Pt/30%CNT-XC72 has better electrochemical durability. With respect to the Pt/20%GO-XC72 (Figure 8E, F), its initial ECSA and MA are 66.1 m^2^·g_Pt_^-1^and 65.8 A g^-1^, and 51.9 m^2^·g_Pt_^-1^and 53.7 A g^-1^ after 5,000 cycles aging, respectively. After 10,000 revolutions of accelerated aging, the ECSA decreases by 29.6%, and the MA decreases by 26.4%, respectively. Compared with the commercial catalyst results, the ECSA is 2.8 times and the MA is 1.7 times higher for Pt/20%GO-XC72, indicating its longer electrochemical durability. The causation can mainly be ascribed to the easy corrosion of carbon black under the acidic and high potential environment, while the graphitic structure of CNTs and GO is comparatively stable. Additionally, due to the strong interaction between the Pt nanoparticles and CNTs or GO, the active phase is less likely to be leached, thus showing better durability.

### 3.3 Oxygen transport resistance

**FIGURE 9:**
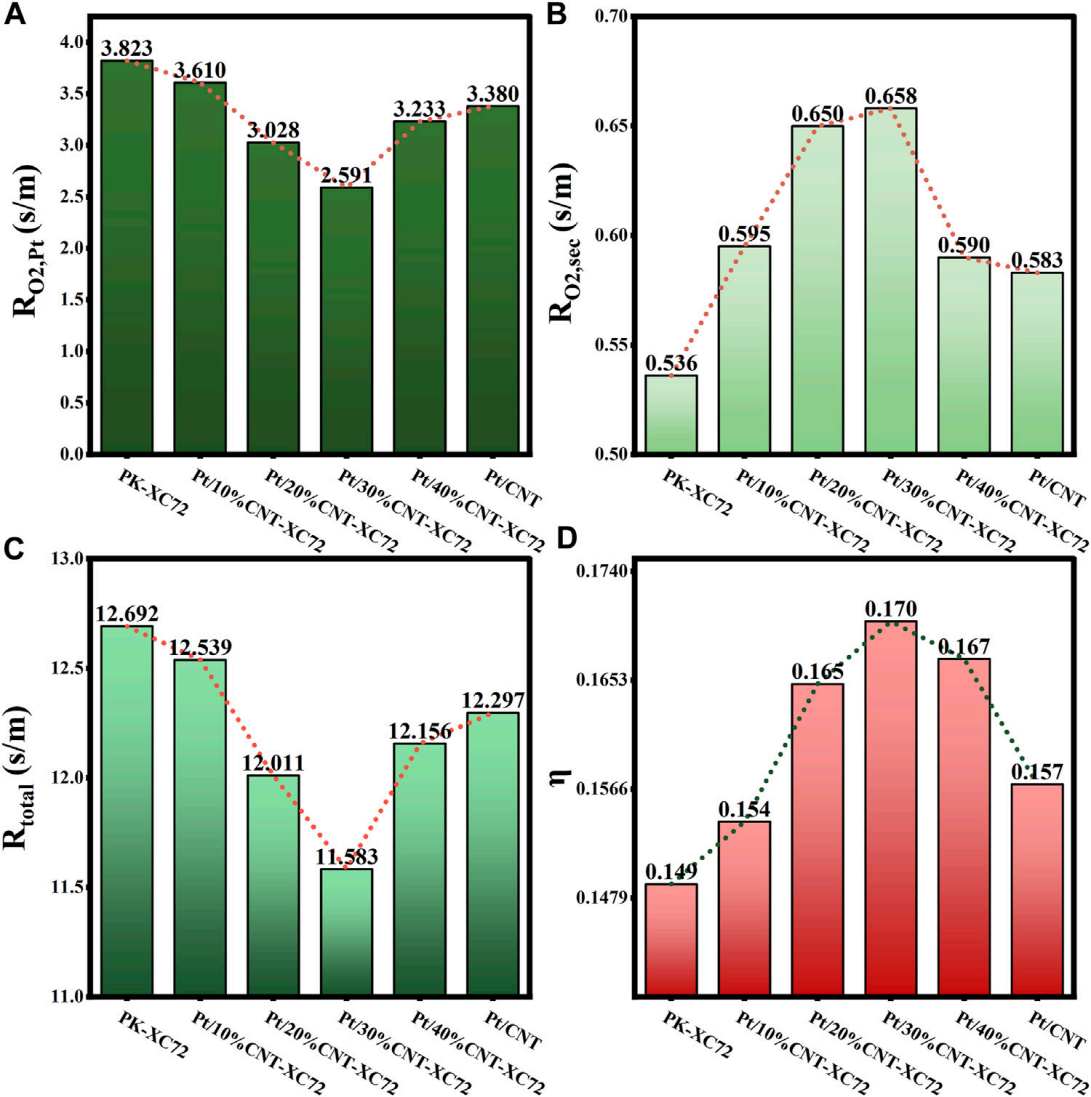
**(A)** Internal oxygen diffusion resistance; **(B)** Oxygen transfer resistance in secondary pores; **(C)** Total oxygen transfer resistance; **(D)** Internal diffusion efficiency factor for Pt/CNT-XC72 series.

**FIGURE 10:**
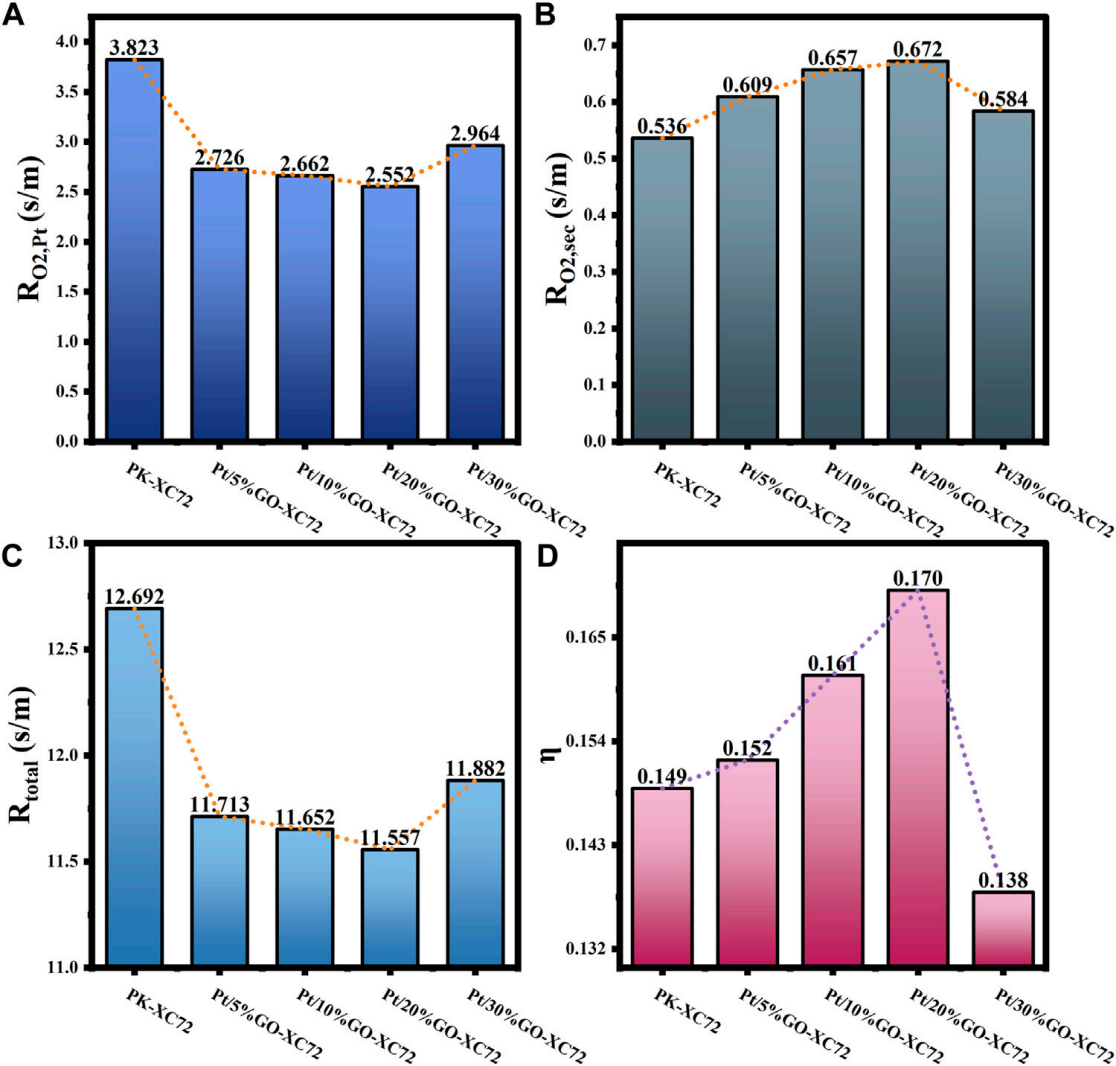
**(A)** Internal oxygen diffusion resistance; **(B)** Oxygen transfer resistance in secondary pores; **(C)** Total oxygen transfer resistance; **(D)** Internal diffusion efficiency factor for Pt/GO-XC72 series.

The results of $R_{O_{2},Pt}$ , $R_{O_{2},\mathrm{Sec}}$ , *R*_total_, and *η* calculations are presented in Figures 9, 10, which demonstrate the influence of respective addition of CNTs and GO on the oxygen mass transfer properties.

As shown in Figure 9, the addition of CNTs results in a decrease and subsequent increase in both $R_{O_{2},Pt}$ and *R*_total_ as the amount of addition increases, with the minimum internal oxygen diffusion resistance ( $R_{O_{2},Pt}$ ) observed at a 30% addition. However, the mass transfer resistance within the secondary pores increases with the addition of CNTs, which can be attributed to the decrease in porosity caused by the filling of CNTs (refer to Eq. 4; Table 1). The total oxygen transfer resistance within the catalytic layer (*R*_total_) varying with the CNT addition amount takes a similar tendency to $R_{O_{2},Pt}$ , suggesting the overwhelming contribution of the latter to the former. As a comprehensive evaluation indicator, the internal diffusion efficiency factor (*η*) maximizes at a 30% addition of CNTs in XC72.

Regarding the impact of GO addition, it can be seen in Figure 10 that the results of $R_{O_{2},Pt}$ and *R*_total_ of the samples involving GO drop significantly compared to those of the commercial PK-XC72 catalyst. However, the $R_{O_{2},\mathrm{Sec}}$ increases with the addition of GO, resembling the effect of CNT addition to XC72. The internal diffusion efficiency factor *η* rises with the adjunction of a small quantity of GO in the catalysts, and reaches the maximum when adding 20%GO into XC72. Obviously, adding appropriately with CNTs or GO can effectively reduce the internal mass transfer resistance of the supported catalysts and improve the overall mass transfer efficiency of the catalysts.

**FIGURE 11:**
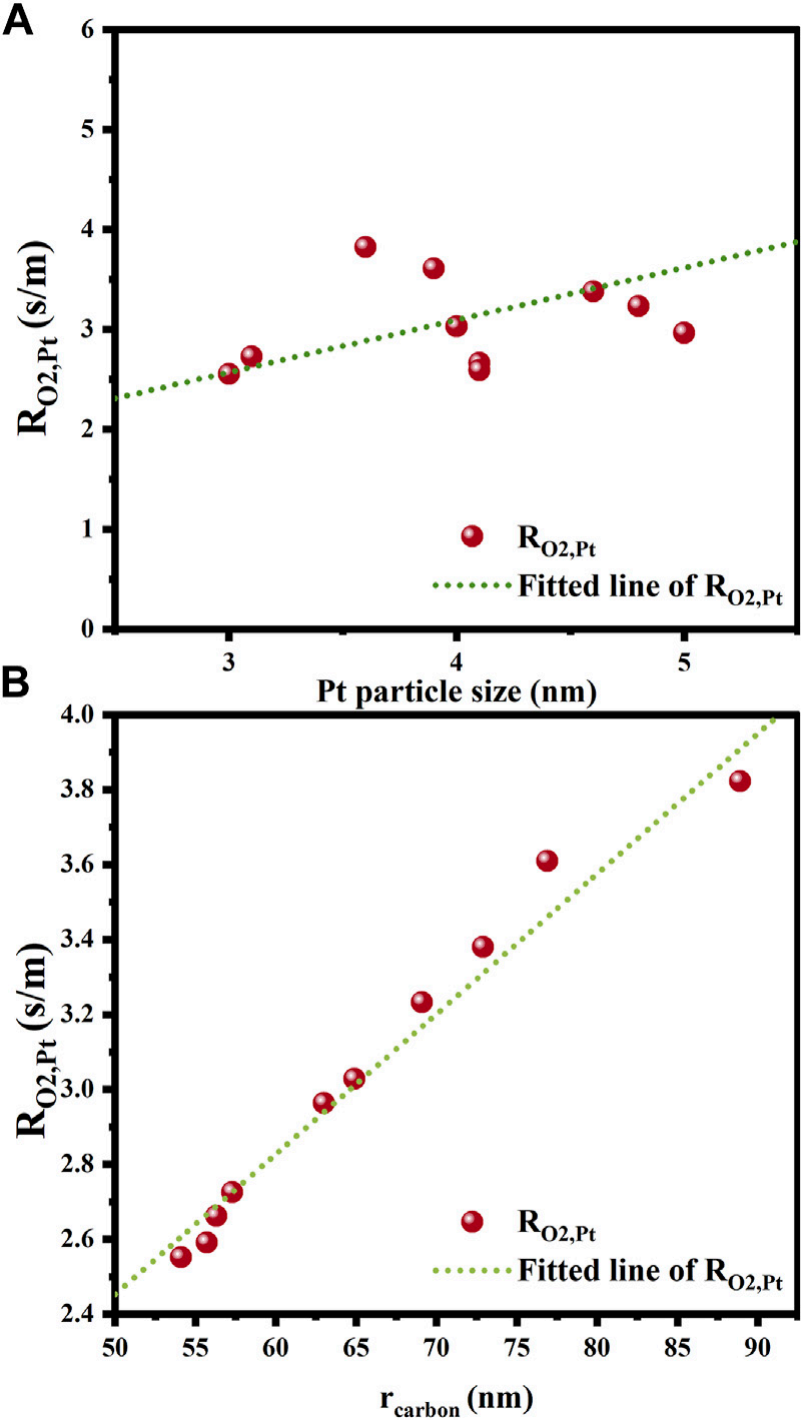
Effects of structural parameters on internal oxygen diffusion resistance. **(A)** Platinum particle size; **(B)** Carbon particle radius.

The correlations of mass transfer resistance and internal diffusion efficiency factor with the structure parameters of all the catalysts studied are established to seek for the dominant structural factors affecting mass transfer resistance in various catalyst layer parts. As depicted in Figure 11, the correlation of $R_{O_{2},Pt}$ with Pt nanoparticle size is positive, which is consistent with the results of previous studies (Sakai et al., 2009; Nonoyama et al., 2011). Within a catalyst layer with a specific platinum loading amount, larger Pt nanoparticles generally give rise to fewer sites exposed and consequently longer mass transfer paths, thus increasing the mass transfer resistance. The particle radius of the carbon material also influences the internal oxygen diffusion resistance. It appears that the carbon particles of larger radius are not conducive to reducing the internal oxygen diffusion resistance, which may be due to the fact that larger carbon particles have a smaller external surface area for the same carbon material mass. As a consequence, the dispersion or the accessibility of the Pt particles deposited becomes worse. By this token, reducing the size of Pt nanoparticles and selecting smaller carbon particles as the supports are effective ways to diminish the internal mass transfer resistance.

**FIGURE 12:**
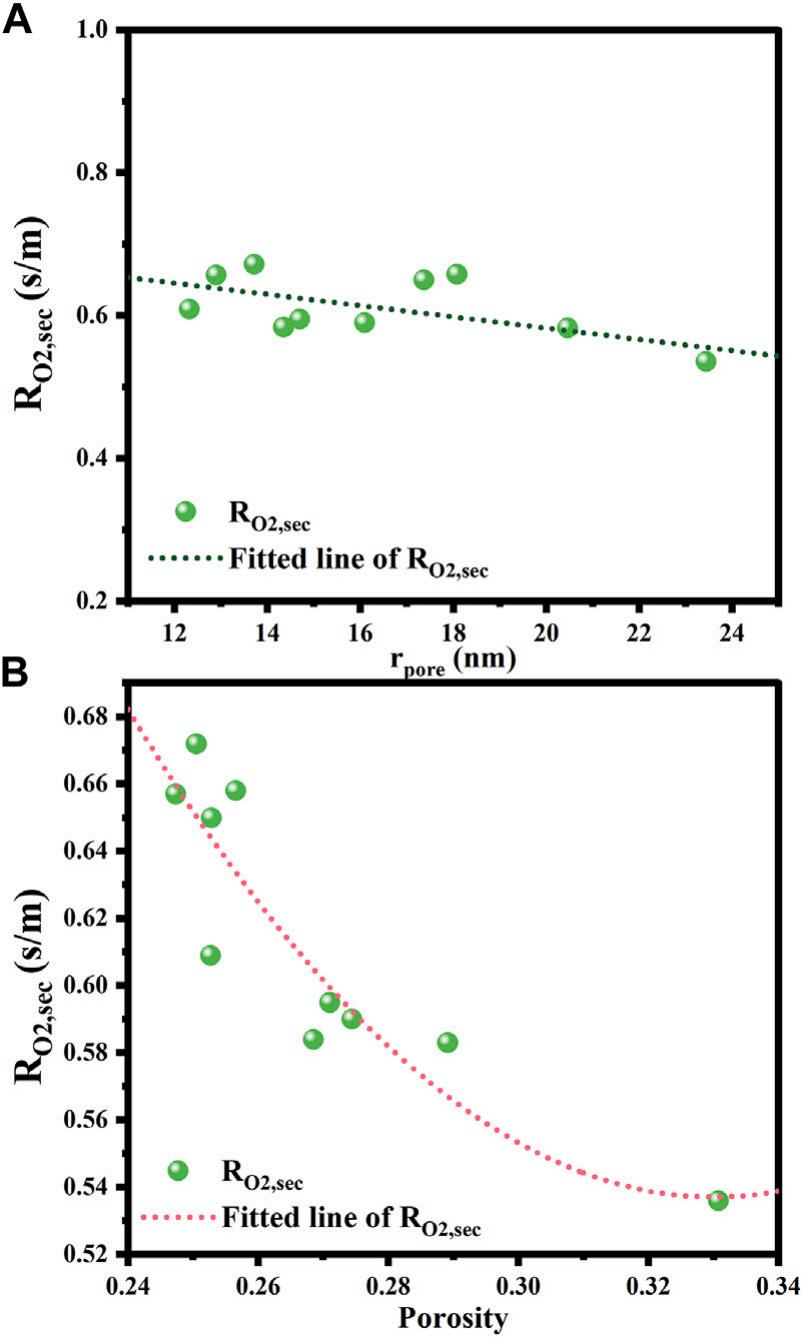
Effects of structural parameters on oxygen transfer resistance and internal diffusion efficiency factor. **(A)** Pore radius; **(B)** Porosity.

Figure 12 illustrates the results of the correlation between the oxygen mass transfer resistance in secondary pores and the pore radius and porosity. From Figure 12A, it can be seen that the oxygen transfer resistance in the secondary pores decreases with increasing the average pore size in the range of 12–24 nm, which is in line with the expectancy that larger pores are more favorable for the diffusion of oxygen molecules. High porosity also benefits the mass transfer through the secondary pores, as proven in Figure 12B. However, it should be noted that if the mass of the CL remains unchanged, the thickness of the CL increases with the mean pore size and porosity. This would instead extend the mass transfer paths within the CL, thus increasing the mass transfer resistance. Therefore, a sparse and porous CL structure can help to reduce the oxygen transfer resistance in the secondary pores only if the CL thickness keeps constant.

## 4 Conclusion

In summary, this study investigated the effects of CL texture on the oxygen transfer capacity and on the ORR performance, both of which are important properties of PEMFC operation especially at high current densities. The integration of theoretical calculations with experimental data demonstrate that incorporating suitable quantity of CNTs or GO into the carbon support can modify the CL texture, thereby facilitating oxygen transfer through the CL.

The supported Pt catalyst layer with 30%CNT or 20%GO addition into XC72 carbon black displays excellent electrochemical performance with 15.8% or 12.1% higher limiting current density, respectively, and higher onset potential and improved electrochemical durability relative to commercial PK-XC72 catalyst. Theoretical calculations verifies the beneficial effect of CL composed of XC72 interweaving with CNT or GO on local oxygen transfer. Furthermore, the smaller particle sized carbon materials with smaller Pt nanoparticles can minimize the internal oxygen transfer resistance. A less dense CL structure at a certain layer thickness can reduce the oxygen transfer resistance within the secondary pores. Therefore, when selecting catalyst supports, consideration should be given to the particle size of the supports and the surface chemistry of the supports for Pt nanoparticles deposition, followed by the porosity and mean pore radius, to enhance the mass transfer towards the Pt nanoparticles and in the secondary pores as well.

## Data Availability

The original contributions presented in the study are included in the article/Supplementary Material, further inquiries can be directed to the corresponding author.
