# Supplementary material for: Textural effect of Pt catalyst layers with different carbon supports on internal oxygen diffusion during oxygen reduction reaction
Source: Front Chem. 2023 Jun 7;11:1217565. doi: 10.3389/fchem.2023.1217565 (PMC10282131; doi:10.3389/fchem.2023.1217565)
Supplement: Supplementary file 1 [file DataSheet1.docx]

Supplementary Material

Textural effect of Pt catalyst layers with different carbon supports on internal oxygen diffusion during oxygen reduction reaction

Wenli Zhang^1^, Zhejie Ma^1^, Xuankai Zhao^1^, Liheng Zhou^1^, Liu Yang^1^, Ping Li^1,^[[1]](#footnote-1)^*^

*** Correspondence:** Ping Li: lipingunilab@ecust.edu.cn


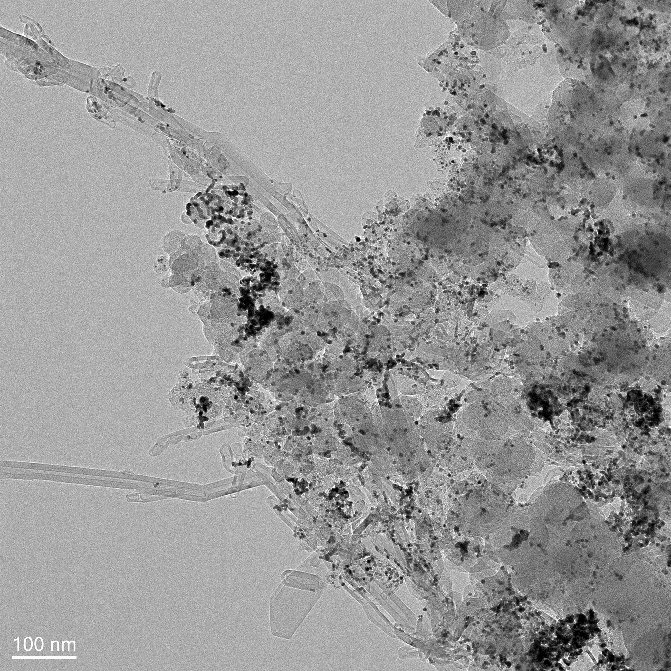

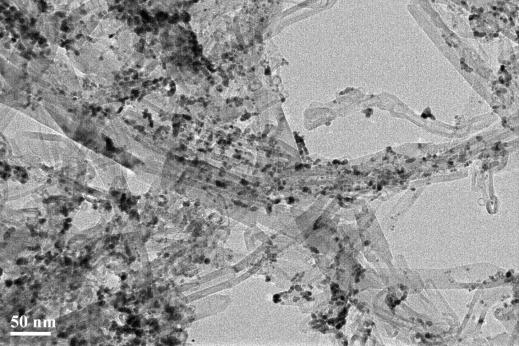

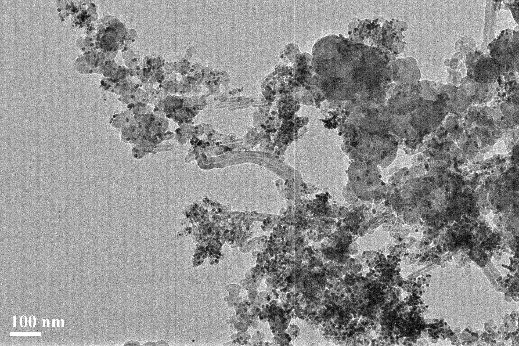


**(A)**

**(B)**


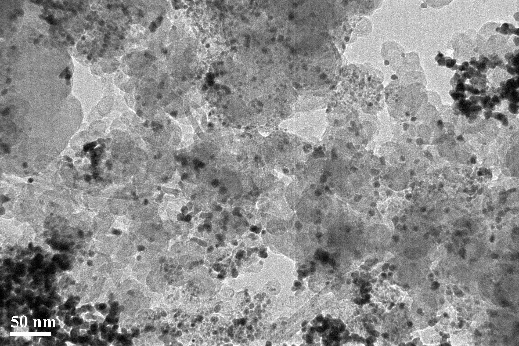

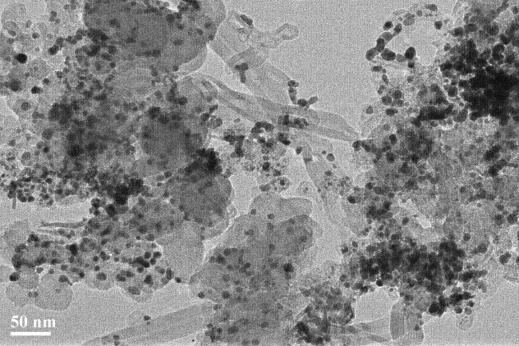


**(C)**

**(D)**


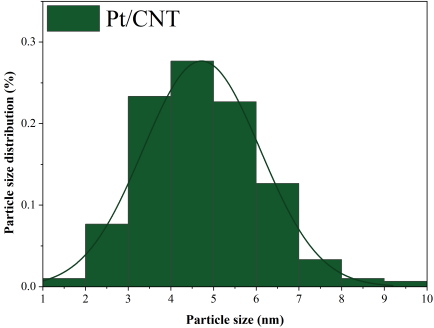

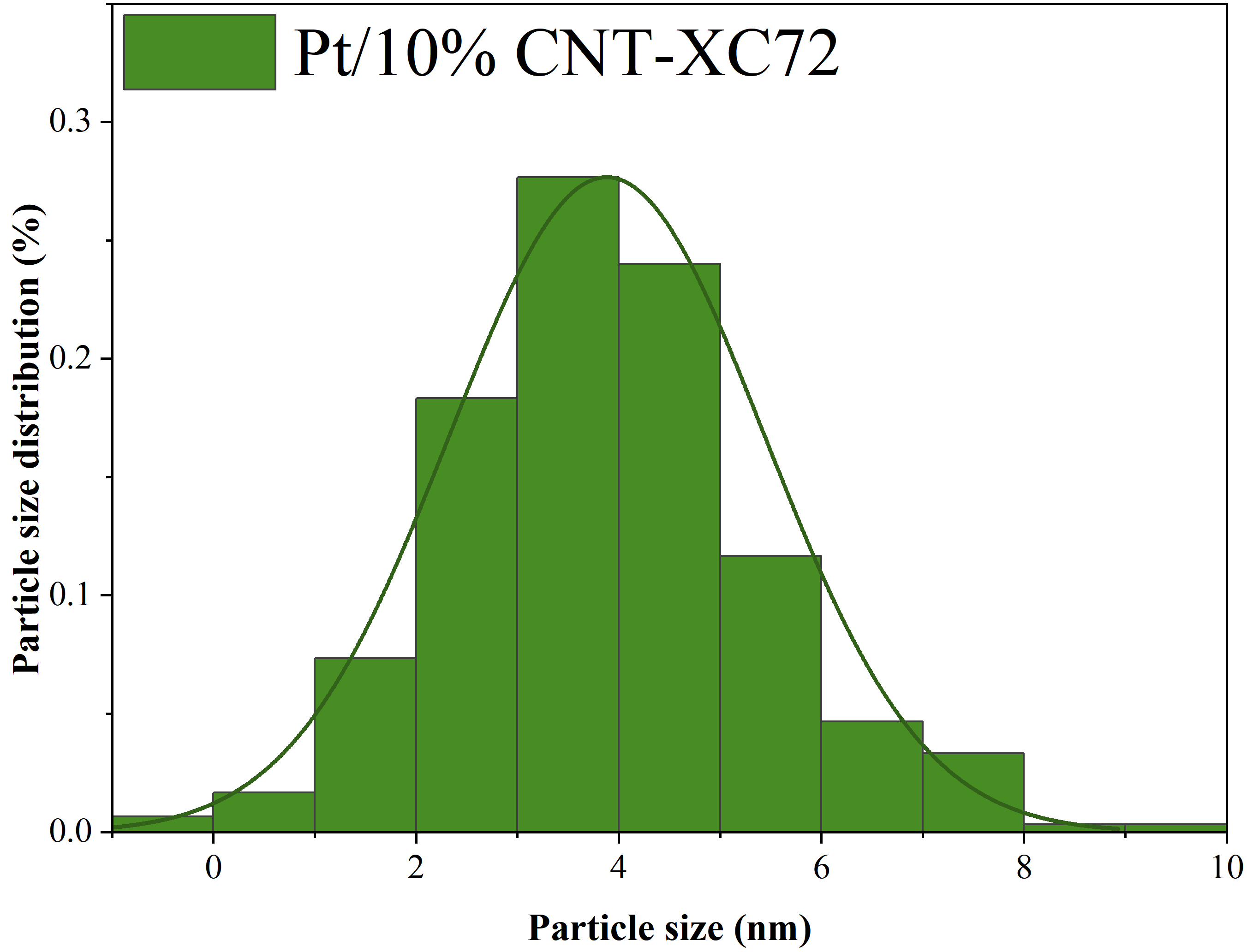

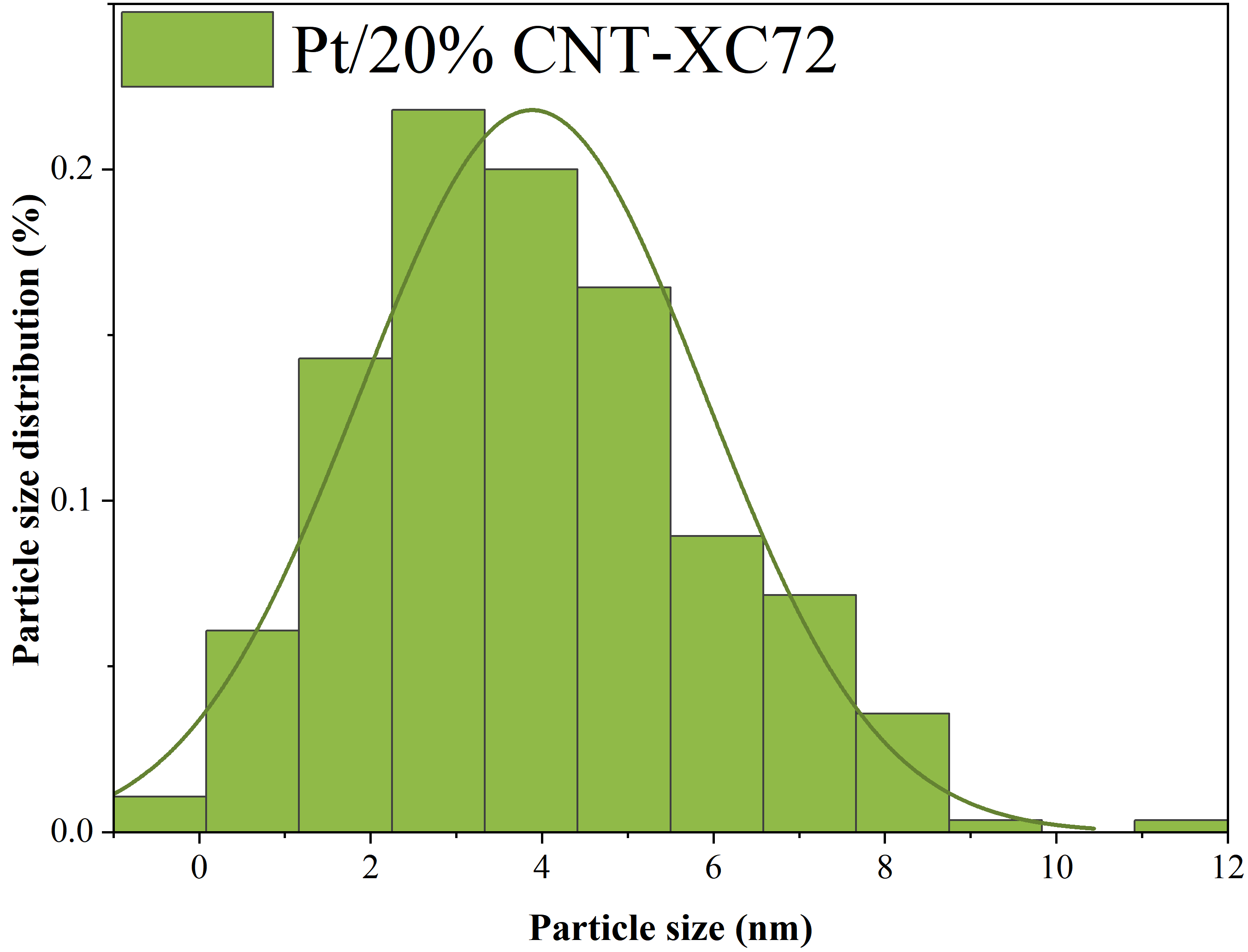

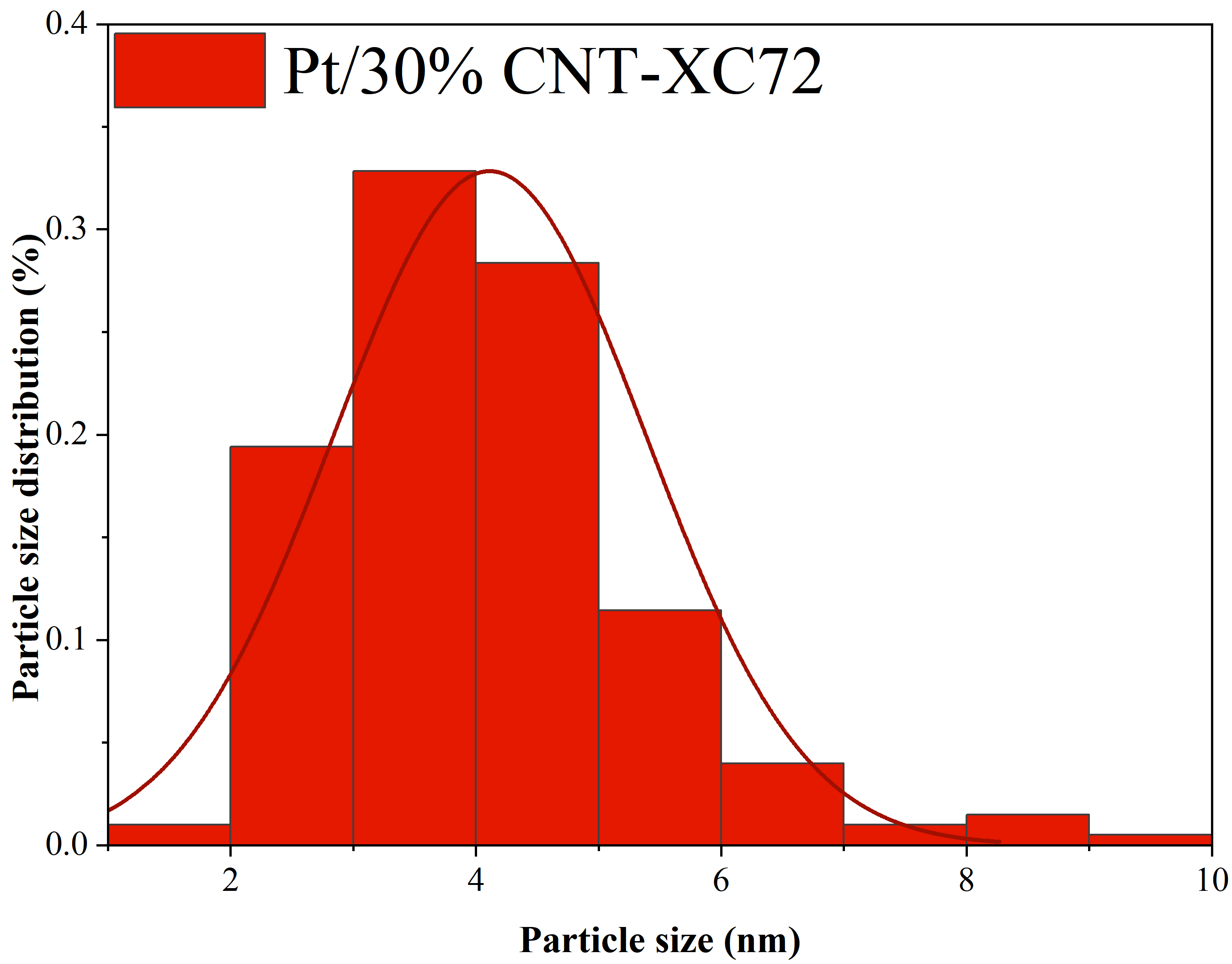

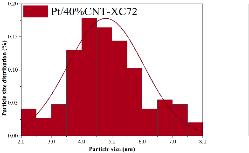


**(E)**


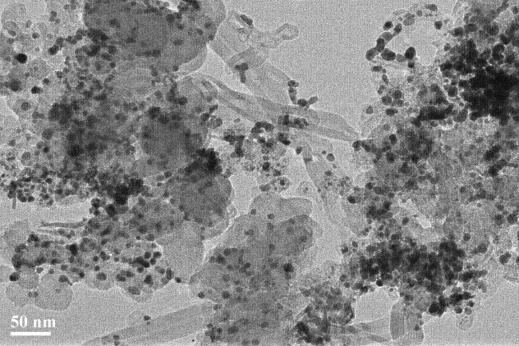

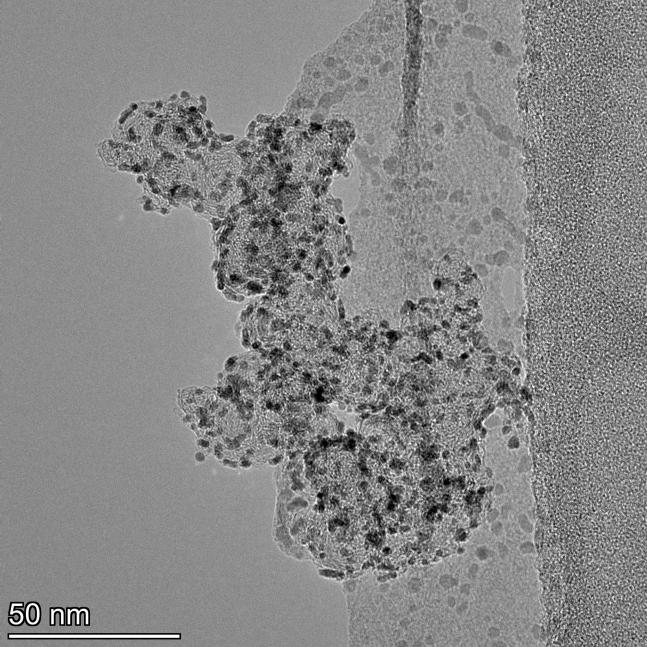

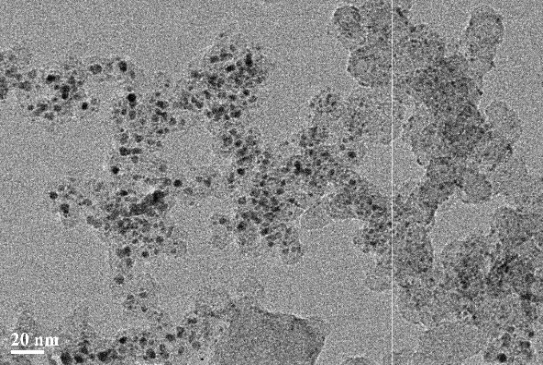


**(F)**

**(G)**


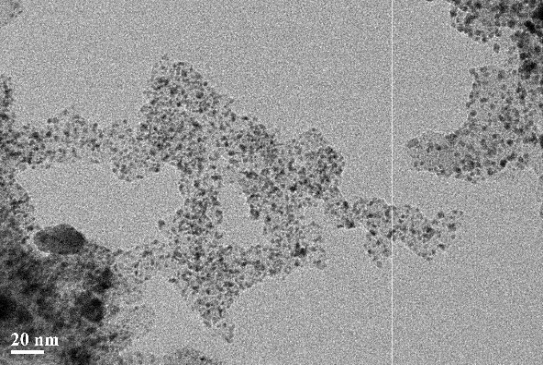

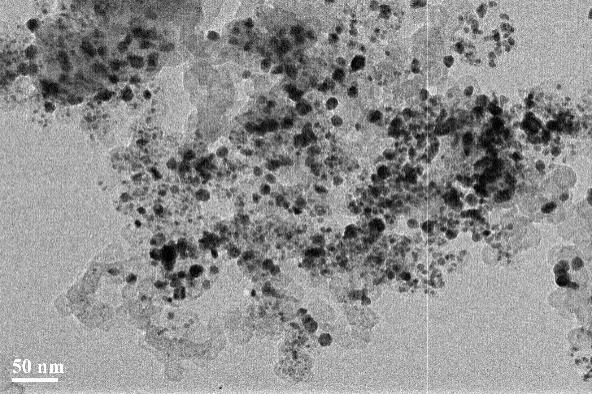


**(H)**

**(I)**


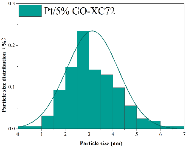

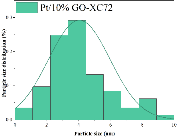

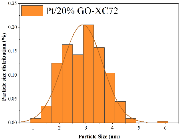

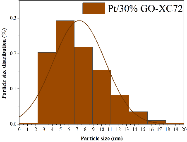

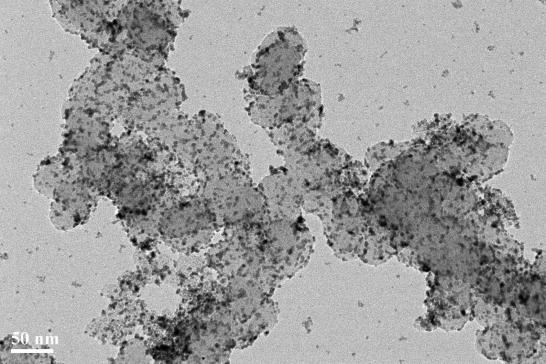

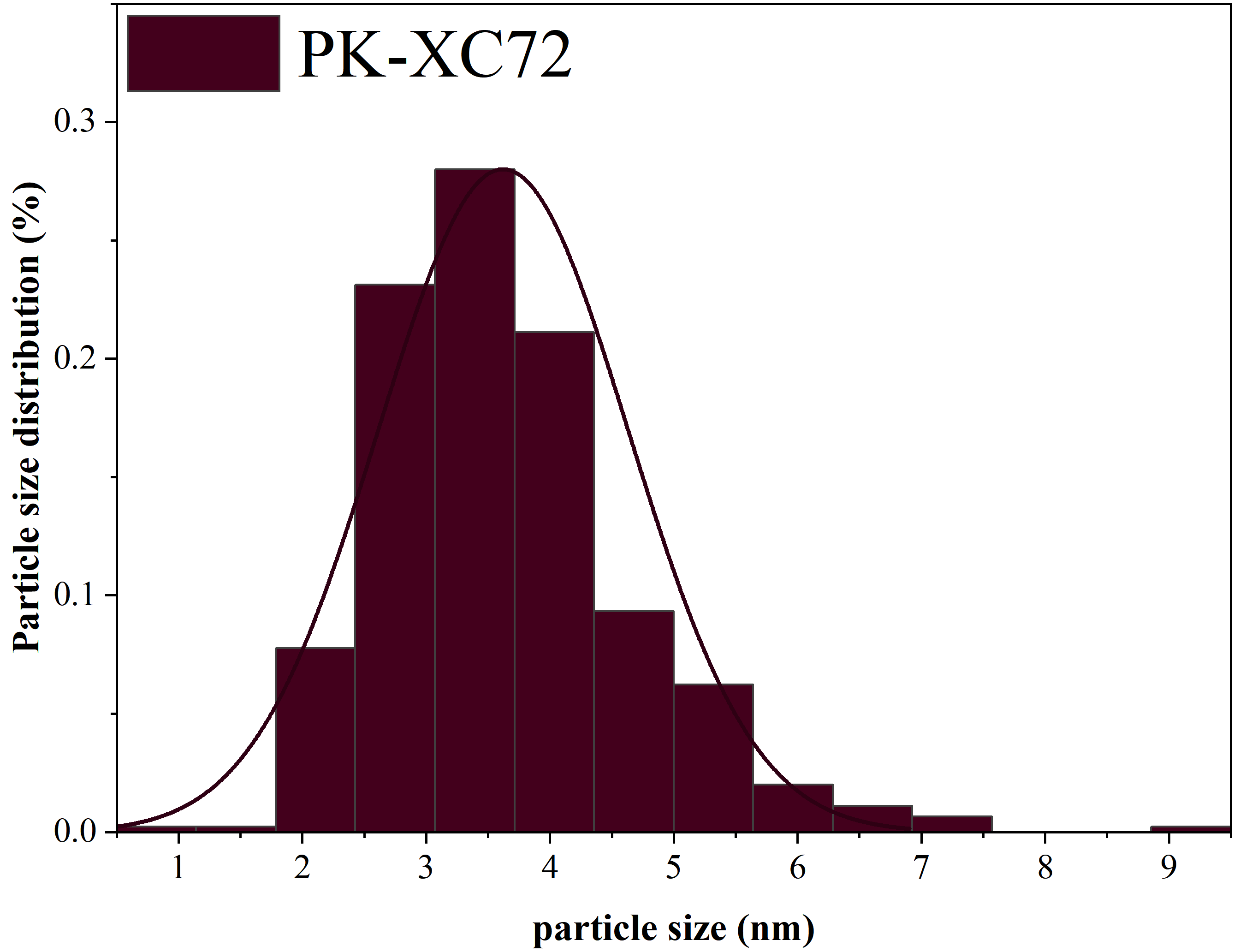


**(J)**


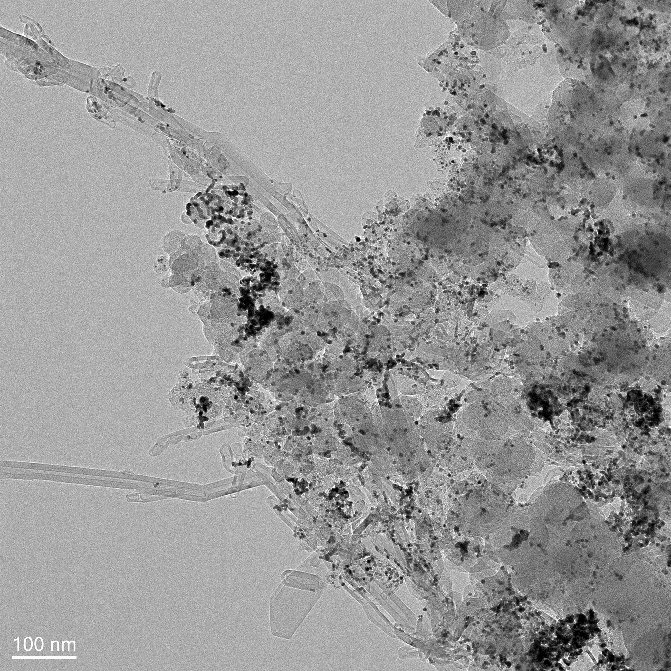

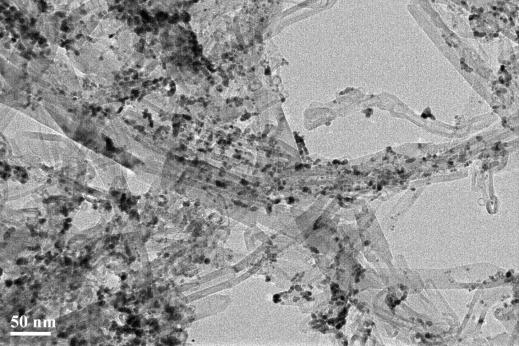

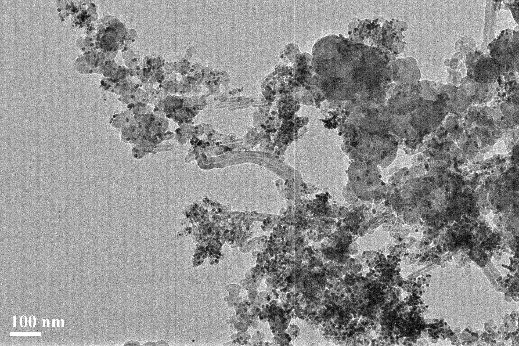


**(A)**

**(B)**


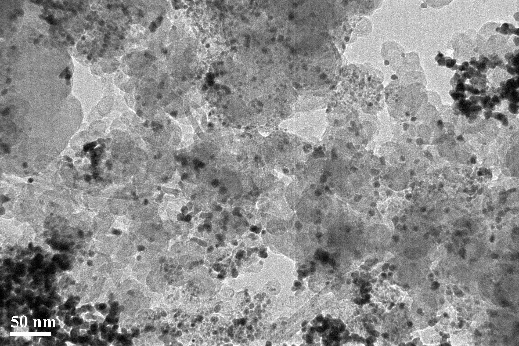

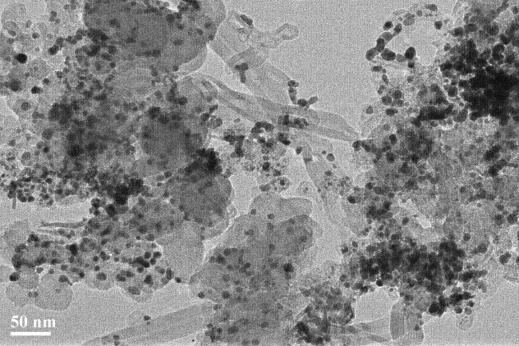


**(C)**

**(D)**


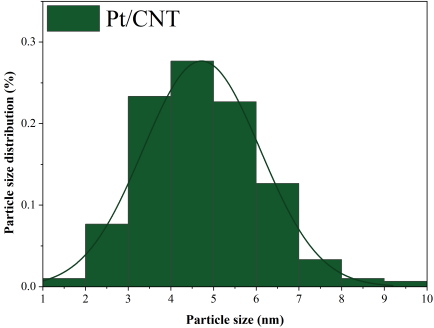

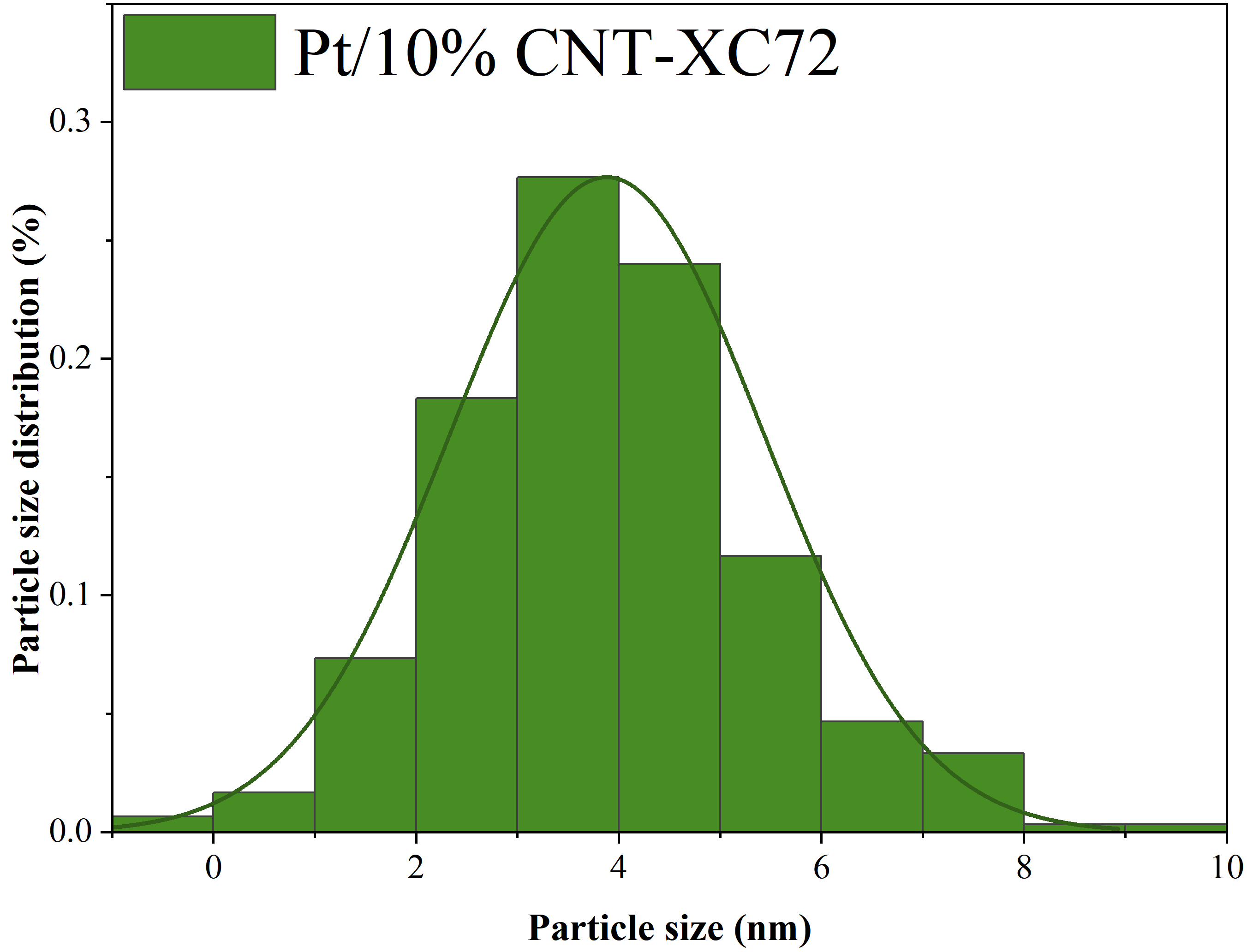

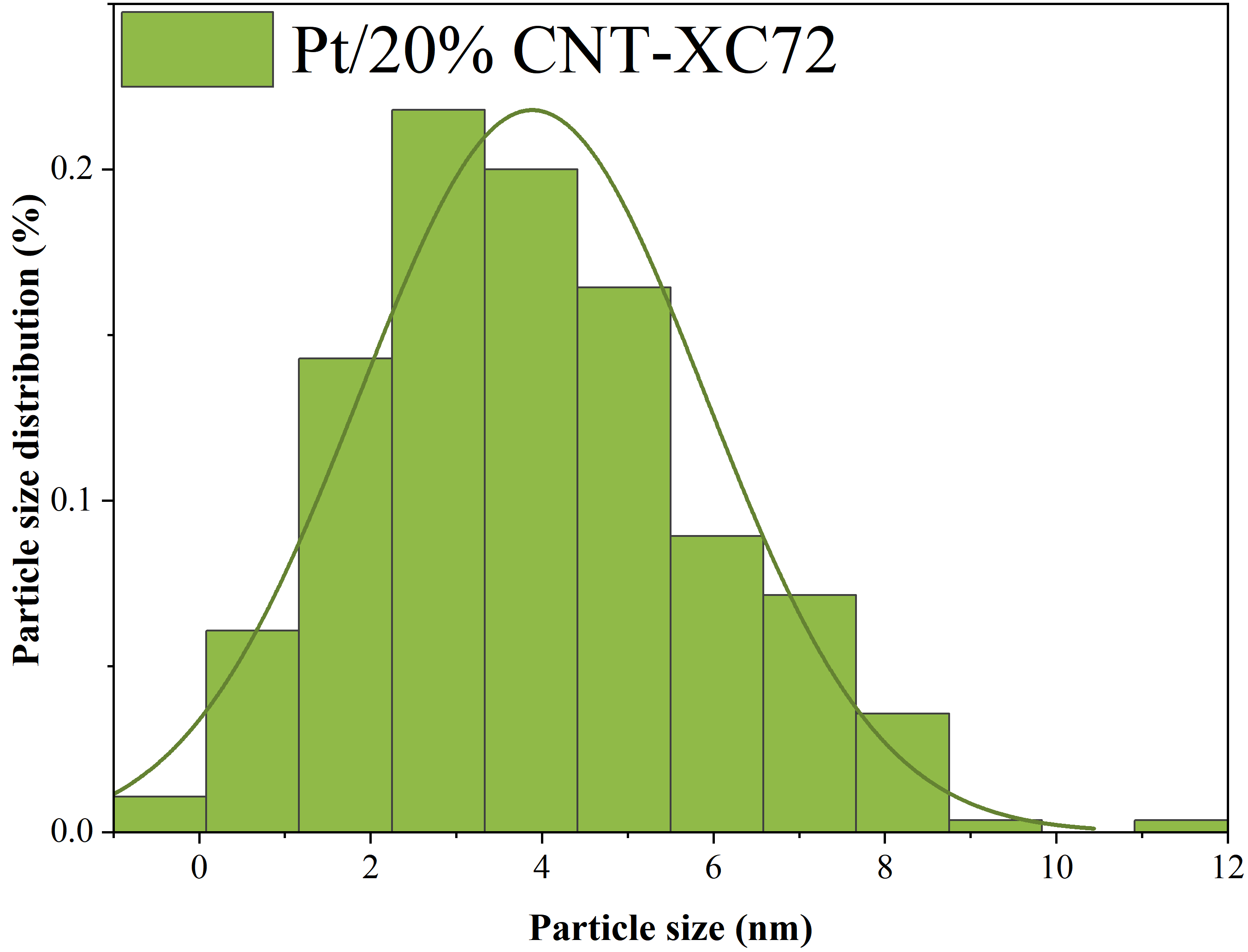

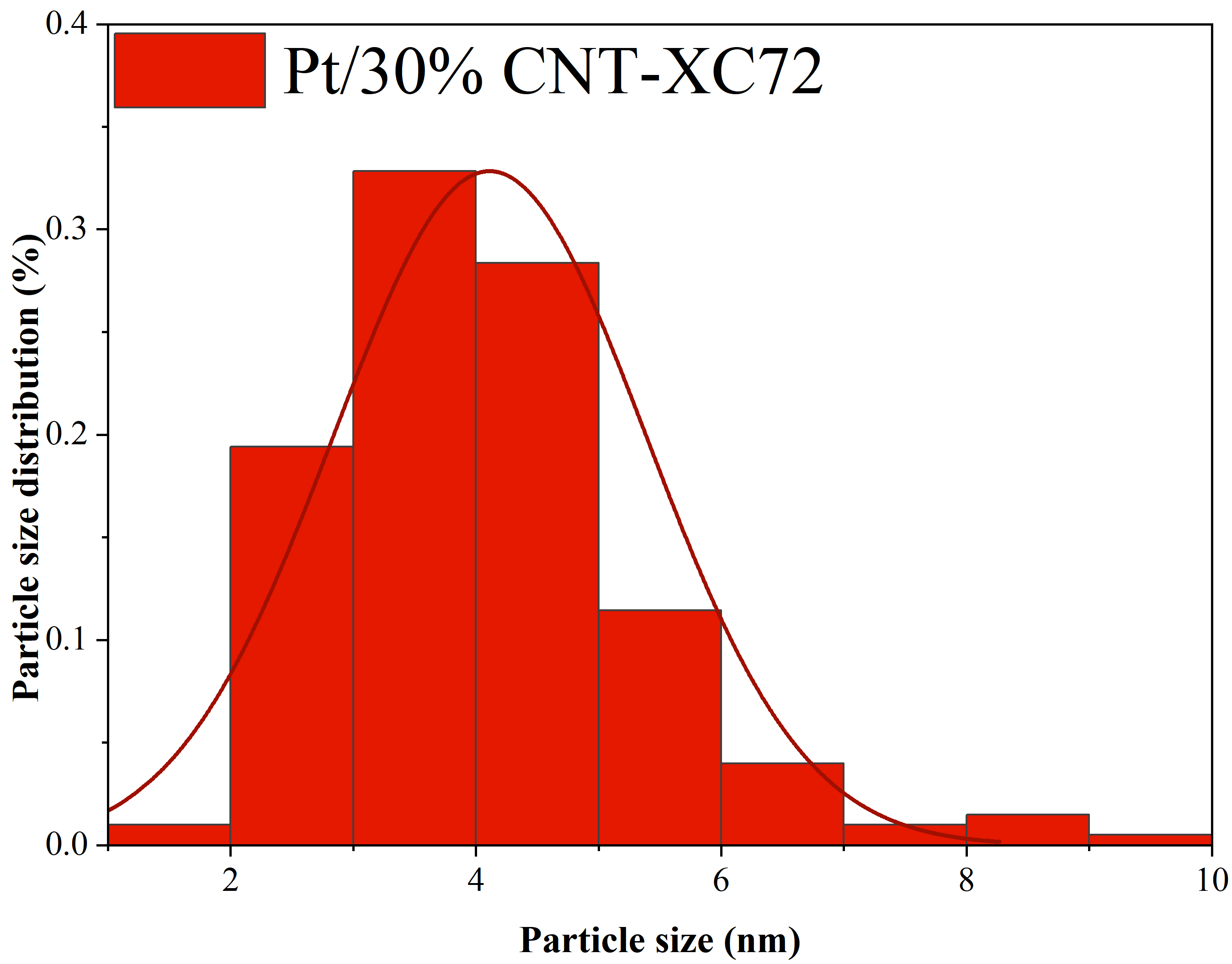

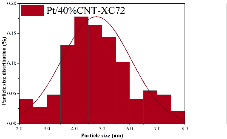


**(E)**


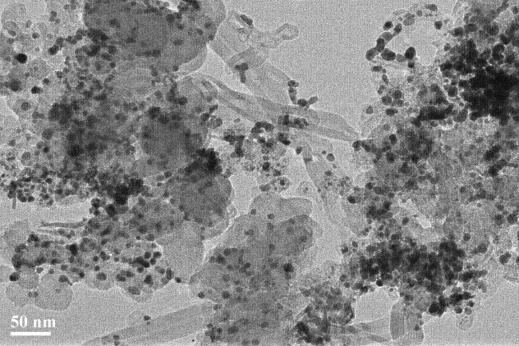

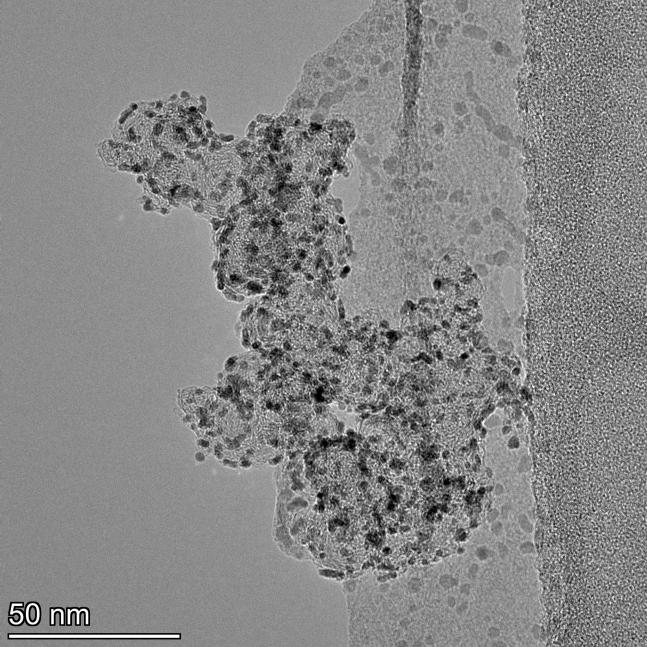

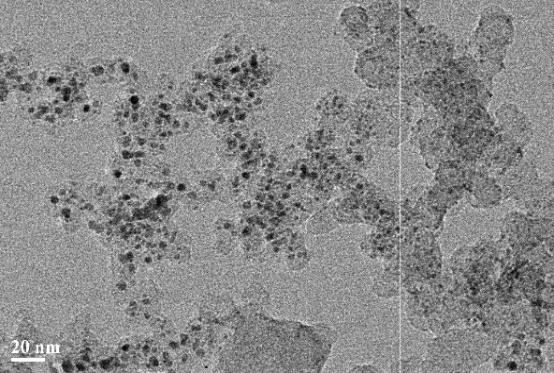


**(F)**

**(G)**


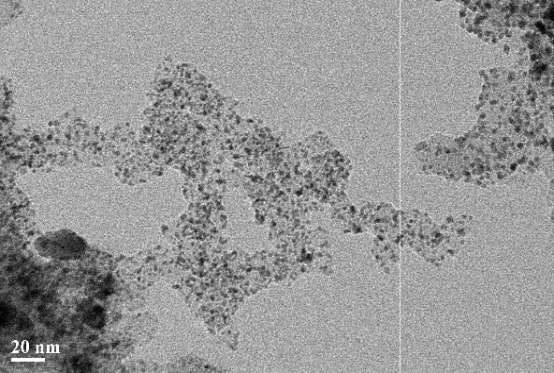

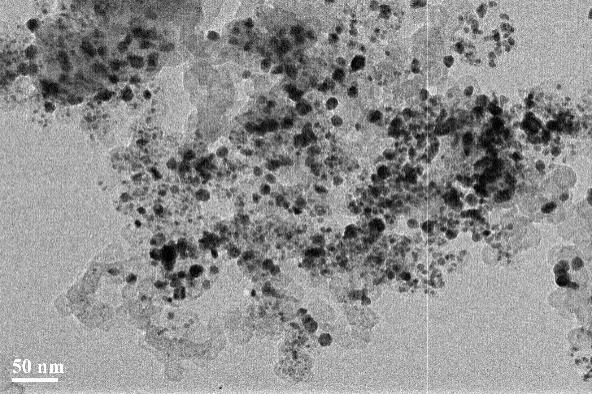


**(H)**

**(I)**


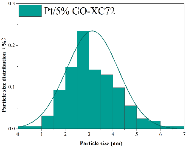

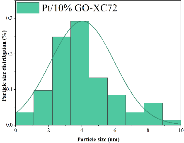

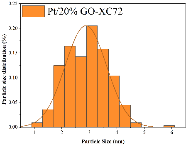

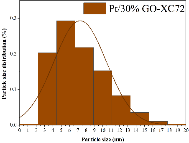

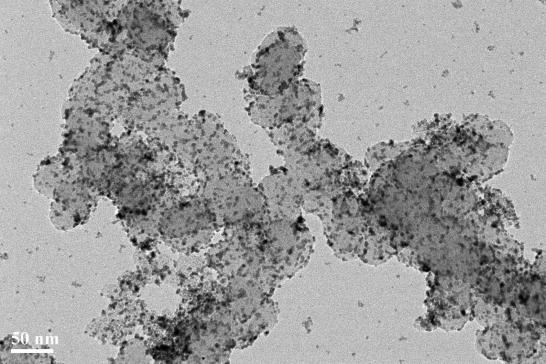

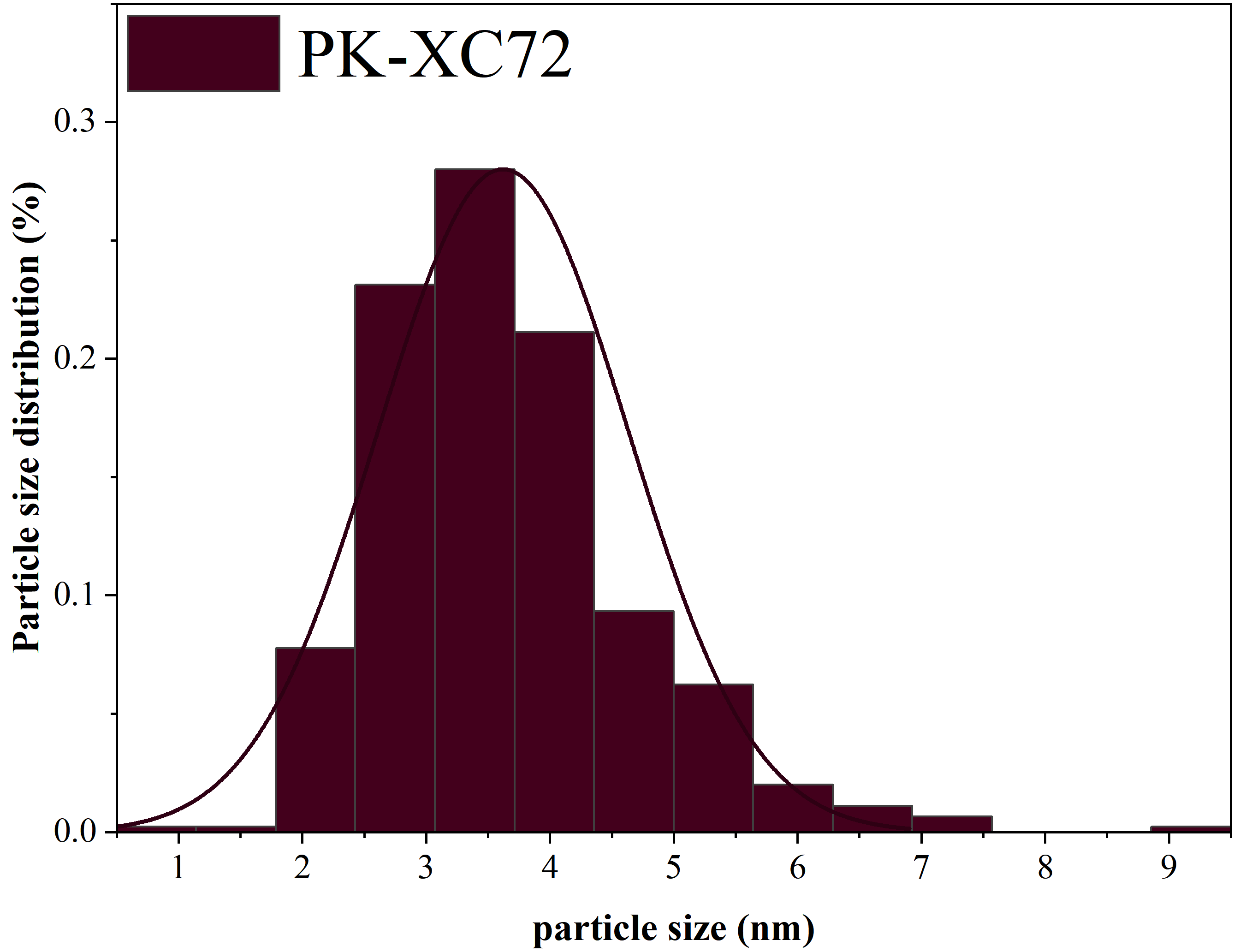


**(J)**

**Figure S1.** HR-TEM images of catalysts (A) Pt/CNT; (B) Pt/10%CNT-XC72; (C) Pt/20%CNT-XC72; (D) Pt/30%CNT-XC72; (E) Pt/40%CNT-XC72; (F) Pt/5%GO-XC72; (G) Pt/10%GO-XC72; (H) Pt/20%GO-XC72; (I) Pt/30%GO-XC72; (J)PK-XC72.


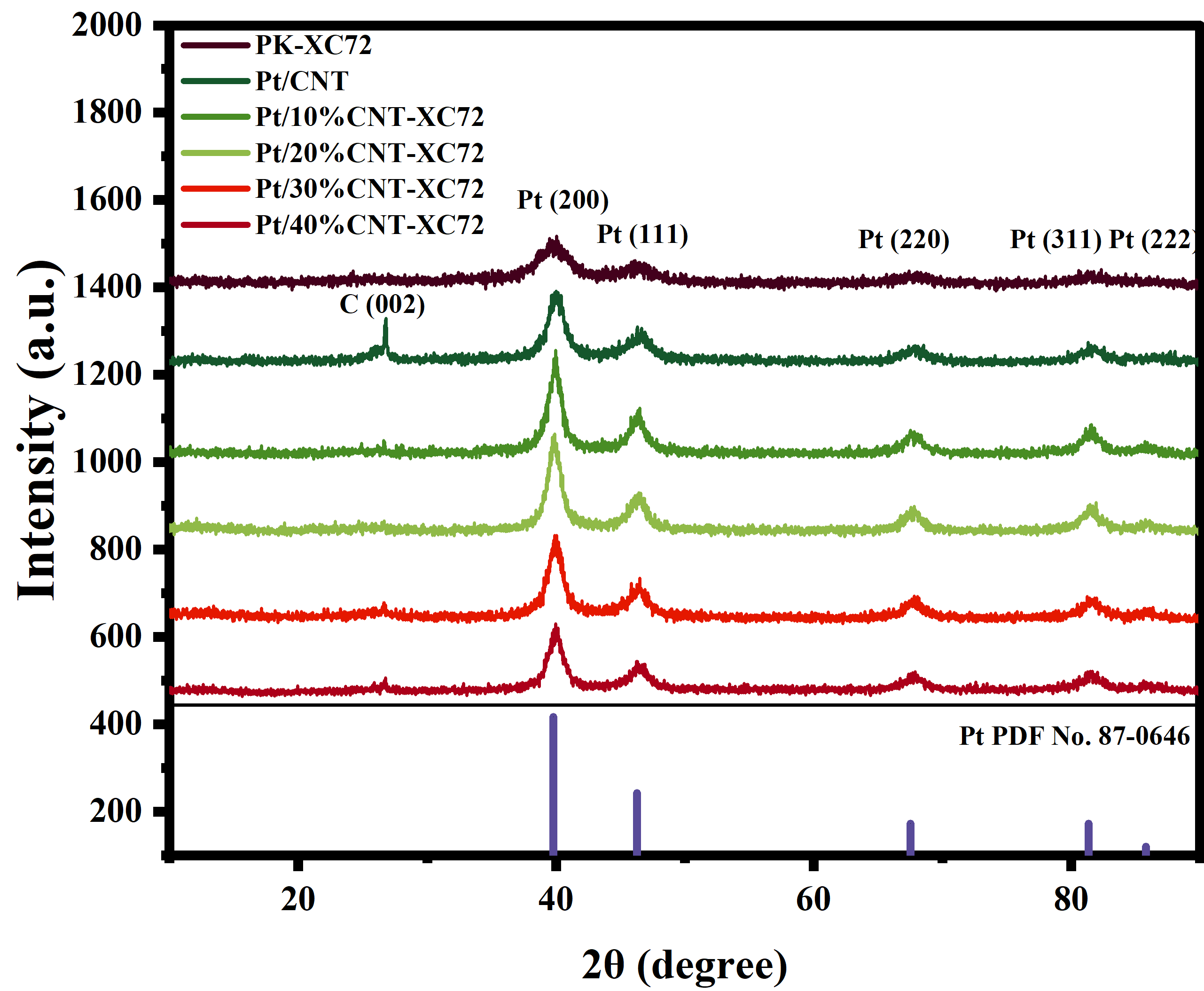


(B)

(A)


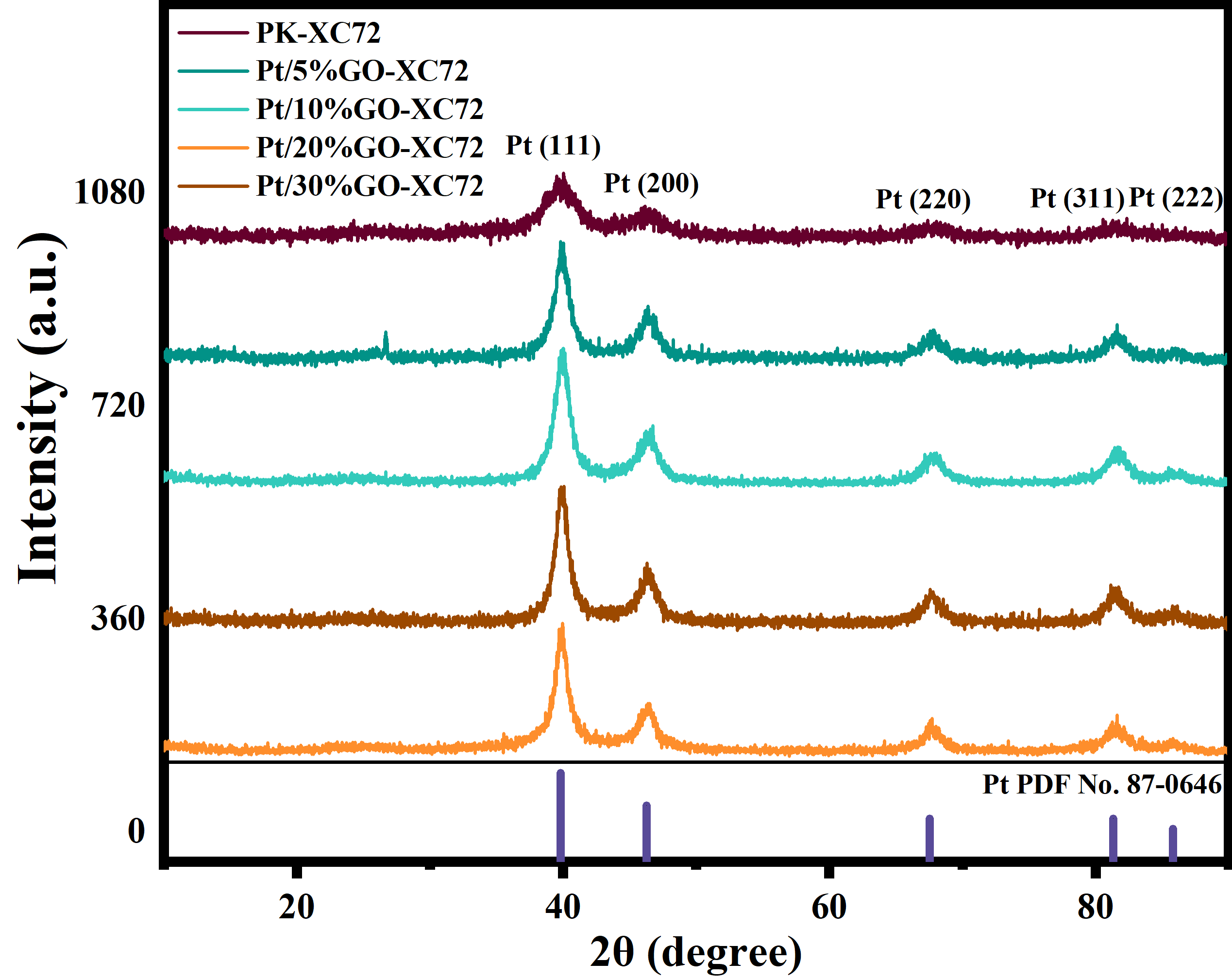


**Figure S2.** XRD diffractograms of catalysts. (A) Pt/CNT-XC72 series; (B) Pt/GO-XC72 series.

1. * Corresponding author. Tel.: +86-13641673447. E-mail address: lipingunilab@ecust.edu.cn (P. Li). [↑](#footnote-ref-1)
